# *Primula nutans* Georgi Extract Inhibits Early Adipogenesis Through CHOP-Associated Regulation and Ameliorates Obesity and Insulin Resistance

**DOI:** 10.3390/ijms27114693

**Published:** 2026-05-22

**Authors:** Nayoung Roh, Kyeoungtae Park, Ducdat Le, Eunbin Kim, Thinhulinh Dang, Thientam Dinh, Badamtsetseg Bazarragchaa, Soo-Yong Kim, Sung-Suk Suh, Jung Jin Kim, Mina Lee, Jong Bae Seo

**Affiliations:** 1 Department of Biomedicine, Health & Life Convergence Sciences, BK21 Four, Biomedical and Healthcare Research Institute, Mokpo National University, Jeonnam 58554, Republic of Korea; youngna5201@naver.com (N.R.); yescom2000@mokpo.ac.kr (K.P.); dmsqls0749@naver.com (E.K.); sungsuksuh@mokpo.ac.kr (S.-S.S.); 2 College of Pharmacy and Research Institute of Life and Pharmaceutical Sciences, Sunchon National University, Jeonnam 57922, Republic of Korea; ddle@scnu.ac.kr (D.L.); 1220173@s.scnu.ac.kr (T.D.); 1243010@s.scnu.ac.kr (T.D.); 3 Department of Natural Cosmetics Science, Sunchon National University, Jeonnam 57922, Republic of Korea; 4 Natural History Museum of Mongolia, Ulaanbaatar 15141, Mongolia; batamtsetseg71@gmail.com; 5 International Biological Material Research Center, Korea Research Institute of Bioscience and Biotechnology, Daejeon 34141, Republic of Korea; soodole@kribb.re.kr; 6 Department of Biosciences, Mokpo National University, Jeonnam 58554, Republic of Korea; jjkim070512@mnu.ac.kr

**Keywords:** adipocyte, differentiation, adipogenesis, *Primula nutans*, obesity, insulin resistance, network pharmacology, molecular docking

## Abstract

*Primula nutans* Georgi, a medicinal herb used in Mongolian and Tibetan medicine for treating respiratory ailments, is a natural agent with antiobesity potential. We investigated the antiobesity and insulin-sensitizing effects of *P. nutans* Georgi extract (PGE) using in vitro and in vivo models. In 3T3-L1 preadipocytes, PGE inhibited adipocyte differentiation and lipid accumulation without cytotoxicity, accompanied by the reduced expression of adipogenic transcription factors (PPARG, C/EBPA, and adiponectin) and lipogenic genes (FASN, SCD1, and ACC), particularly during the early stages of adipogenesis. Similar effects were observed in primary stromal vascular cells derived from mouse inguinal white adipose tissue. PGE upregulated C/EBP homologous protein and C/EBPB and was associated with altered cell cycle progression, increased G2/M phase distribution, and the potential disruption of mitotic clonal expansion during early adipogenesis. In HFD-induced obese mice, intraperitoneal administration of PGE (10 or 30 mg/kg) significantly reduced body weight gain, white adipose tissue mass, and hepatic steatosis, independent of food intake. PGE downregulated lipogenic and proinflammatory gene expression in adipose and hepatic tissues and increased AMPK phosphorylation in white adipose tissue. PGE improved glucose tolerance and was associated with enhanced insulin sensitivity, as evidenced by reduced areas under the curve in the glucose tolerance and insulin tolerance tests and increased circulating adiponectin levels. Feature-based molecular networking identified 61 compounds from PGE. Network pharmacology analysis revealed several antiobesity targets, including PPARG and AKT1. Molecular docking analyses suggested favorable binding affinities between major compounds and metabolic regulators. Collectively, these findings suggest that PGE may suppress adipogenesis and improve metabolic parameters in obese mice, supporting its potential as a natural candidate for obesity and related metabolic disorders.

## 1. Introduction

*Primula nutans* Georgi, a perennial herbaceous plant belonging to the *Primulaceae* family, is native to alpine regions of Eurasia and has long been employed in traditional Mongolian and Tibetan medicine for treating respiratory ailments, including bronchitis and pulmonary tuberculosis [1]. Ethnopharmacological records document its use in alleviating inflammatory conditions such as edema and skin infections [1]. Although the anti-inflammatory and antimicrobial properties of related species, such as *Primula veris*, have been investigated [2,3], the specific metabolic effects of *P. nutans* are largely unexplored.

Adipose tissue plays a critical role in maintaining metabolic homeostasis by regulating energy storage and endocrine function [4]. Adipocytes store excess nutrients as triglycerides and secrete adipokines, such as leptin and adiponectin, that influence systemic insulin sensitivity and inflammatory pathways [5,6]. In the context of obesity, however, hypertrophied adipocytes contribute to chronic low-grade inflammation and altered hormone signaling, ultimately leading to insulin resistance and the progression of type 2 diabetes [7]. Visceral fat accumulation is particularly associated with metabolic complications such as nonalcoholic fatty liver disease and cardiovascular disorders [8].

Adipocyte differentiation, or adipogenesis, is a highly coordinated process in which preadipocytes acquire the morphological and biochemical characteristics of mature adipocytes [9]. Adipogenesis is driven by key transcription factors, notably peroxisome proliferator-activated receptor gamma (PPARG) and CCAAT/enhancer-binding protein alpha (C/EBPA), which activate the expression of genes involved in lipid metabolism and adipokine secretion [9,10]. Mitotic clonal expansion (MCE), an early phase of adipogenesis, enables preadipocytes to re-enter the cell cycle prior to terminal differentiation [11]. Inhibition of MCE disrupts adipogenesis and is a potential target for antiobesity interventions [12]. Moreover, cellular stress signaling pathways, such as those mediated by C/EBP homologous protein (CHOP), are increasingly recognized for their roles in modulating adipogenic commitment and metabolic adaptation [13,14].

Given the growing interest in plant-derived bioactives in managing metabolic disorders, several natural products have been identified as regulators of adipocyte differentiation and insulin sensitivity [15,16,17]. However, despite its traditional medicinal use, the antiobesity mechanisms and metabolic regulatory effects of *P. nutans* Georgi extract (PGE), particularly its influence on early adipogenesis and insulin resistance, remain largely unexplored.

In addition to experimental approaches, in silico network pharmacology and molecular docking analyses were employed to identify the putative obesity-related targets of major PGE metabolites and to elucidate its potential multitarget mechanisms of action [18,19]. These computational predictions support experimental findings and provide a mechanistic framework linking the metabolic benefits of PGE to specific molecular interactions.

In this study, we investigated the antiobesity and insulin-sensitizing effects of PGE in both in vivo and in vitro models along with in silico approaches. Using a high-fat diet (HFD)-induced obese mouse model, we evaluated the effects of PGE on body weight gain, lipid accumulation in hepatic and adipose tissues, and glucose homeostasis. Concurrently, we examined the effects of PGE on adipocyte differentiation in 3T3-L1 preadipocytes and primary stromal vascular cells, with a focus on early-stage regulatory mechanisms, including MCE, CHOP expression, and cell cycle progression. These integrative approaches aimed to elucidate the molecular basis of PGE’s metabolic benefits and assess its potential as a natural therapeutic candidate for obesity and related metabolic disorders.

## 2. Results

### 2.1. PGE Inhibits 3T3-L1 Adipocyte Differentiation Without Cytotoxicity

**Figure 1:**
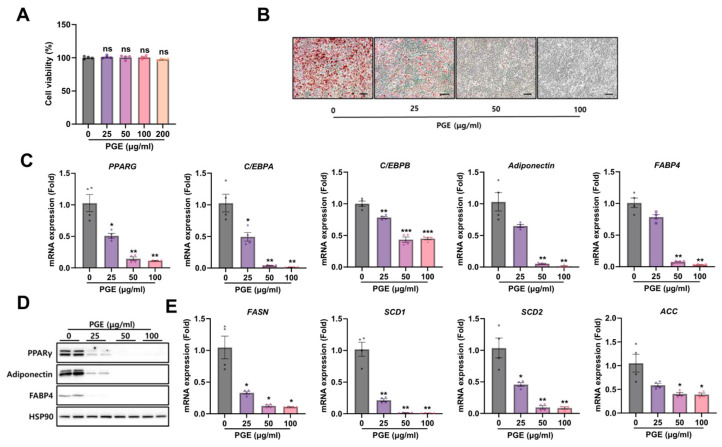
*Primula nutans* Georgi extract (PGE) suppresses adipocyte differentiation and lipogenesis in 3T3-L1 cells. (**A**) Cell viability was assessed using the WST-8 assay in 3T3-L1 preadipocytes treated with various concentrations of PGE (0, 25, 50, 100, and 200 µg/mL). (**B**) Oil red O staining of 3T3-L1 adipocytes differentiated in the presence or absence of PGE (0, 25, 50, and 100 µg/mL) for 6 d. Scale bar = 200 µm; magnification = ×100. (**C**) mRNA expression levels of adipogenic markers (PPARG, C/EBPA, C/EBPB, adiponectin, and FABP4) were quantified by qRT-PCR. (**D**) Protein expression levels of PPARG, adiponectin, and FABP4 were evaluated by Western blotting. HSP90 was used as a loading control. (**E**) mRNA levels of lipogenic genes (FASN, ACC, SCD1, and SCD2) were measured by qRT-PCR. All values represent the mean ± SEM of two independent experiments (*n* = 4). Statistical significance: * *p* < 0.05, ** *p* < 0.01, and *** *p* < 0.001 vs. control (0 µg/mL). Each colored dot represents an individual data point. ns, not significant.

Cytotoxicity assessment using the WST-8 assay showed that various concentrations of PGE (0, 25, 50, 100, and 200 µg/mL) were noncytotoxic to 3T3-L1 preadipocytes. No significant differences in cell viability were observed compared with the untreated controls at any concentration tested (Figure 1A). To evaluate the effects of PGE on adipogenesis, 3T3-L1 cells were treated with PGE during differentiation. Oil red O staining revealed a dose-dependent reduction in lipid accumulation at 25, 50, and 100 μg/mL of PGE (Figure 1B). Consistently, qRT-PCR analysis demonstrated that mRNA expression of key adipogenic markers, including PPARG, C/EBPA, C/EBPB, adiponectin, and FABP4, was significantly downregulated, particularly at 50 and 100 μg/mL of PGE (Figure 1C). Western blotting confirmed a dose-dependent decrease in the protein levels of PPARG, adiponectin, and FABP4 (Figure 1D). PGE suppressed the expression of lipogenesis-related genes—mRNA levels of fatty acid synthase (FASN), stearoyl-CoA desaturase 1 (SCD1), SCD2, and ACC were significantly reduced in a dose-dependent manner, with reductions ranging from 2.5- to 5.2-fold at higher concentrations (Figure 1E). Collectively, these findings suggest that PGE may inhibit adipocyte differentiation, accompanied by reduced adipogenic and lipogenic gene expression and decreased lipid droplet formation, as indicated by both molecular and morphological changes.

### 2.2. PGE Downregulates Adipogenesis Primarily During the Early Stage of Differentiation

**Figure 2:**
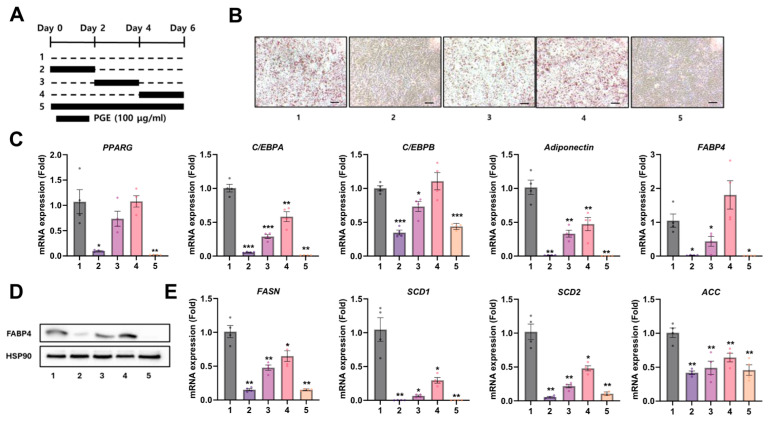
*Primula nutans* Georgi extract (PGE) exerts stage-specific inhibitory effects during adipocyte differentiation. (**A**) Schematic diagram of the treatment schedule indicating the timing of PGE (100 µg/mL) administration during adipocyte differentiation. (**B**) Lipid accumulation assessed by Oil red O staining after 6 d of differentiation. Scale bar = 200 µm; magnification = ×100. (**C**) mRNA expression of adipogenic genes was determined by qRT-PCR. (**D**) FABP4 expression levels were analyzed by Western blotting; HSP90 was used as a loading control. (**E**) Expression of lipogenesis-related genes (FAS, ACC, SCD1, and SCD2) was quantified by qRT-PCR. Data are presented as mean ± SEM from two independent experiments (*n* = 4). Statistical significance: * *p* < 0.05, ** *p* < 0.01, and *** *p* < 0.001 vs. control group. Each colored dot represents an individual data point.

To investigate the stage-specific effects of PGE on adipogenesis, 3T3-L1 cells were treated with 100 μg/mL of PGE during different time windows: early (D0–D2), mid (D2–D4), late (D4–D6), or continuously (D0–D6) (Figure 2A). Oil red O staining revealed that lipid accumulation was significantly reduced when PGE was administered either during the early stage or throughout differentiation (Figure 2B). qRT-PCR analysis showed that the mRNA expression of key adipogenic markers—PPARG, C/EBPA, adiponectin, and FABP4—was reduced by >3-fold in these groups (Figure 2C). Western blotting confirmed that the level of FABP4, a key marker of adipocyte maturation, was markedly decreased when PGE was applied during early differentiation, compared with during mid or late treatment (Figure 2D). Consistently, the expression of lipogenesis-related genes, including FASN, SCD1, SCD2, and ACC, was significantly downregulated in both the early and continuous PGE treatment groups compared with the controls (Figure 2E). Collectively, these findings suggest that PGE may exert anti-adipogenic effects predominantly during the early phase of differentiation, highlighting the potential importance of treatment timing in modulating adipogenesis.

### 2.3. PGE Inhibits Adipogenesis in Primary Preadipocytes Derived from iWAT

**Figure 3:**
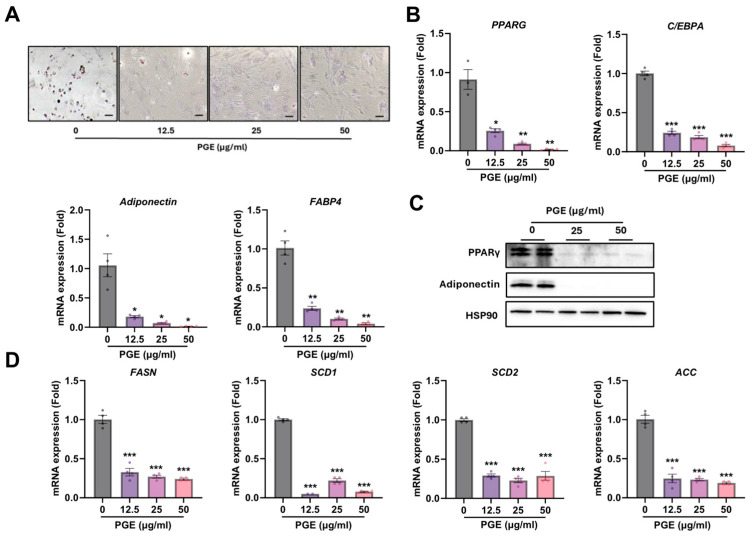
*Primula nutans* Georgi extract (PGE) inhibits adipogenesis and lipogenesis in primary stromal vascular cells (SVCs). (**A**) Oil red O staining of SVCs isolated from inguinal white adipose tissue (iWAT) and differentiated for 6 d with or without PGE (12.5, 25, and 50 µg/mL). Scale bar = 100 µm. (**B**) qRT-PCR analysis of adipogenic gene expression (PPARG, C/EBPA, adiponectin, and FABP4) in SVCs treated with the indicated PGE concentrations. (**C**) Western blotting to evaluate PPARG and adiponectin levels; HSP90 was used as a loading control. (**D**) Expression levels of lipogenesis-related genes (FAS, ACC, and SCD1) were quantified by qRT-PCR. All results are presented as the mean ± SEM from two independent experiments (*n* = 4). Statistical significance: * *p* < 0.05, ** *p* < 0.01, *** *p* < 0.001 vs. control group. Each colored dot represents an individual data point.

To further validate the antiadipogenic effects of PGE in a physiologically relevant model, stromal vascular cells were isolated from mouse inguinal white adipose tissue (iWAT) and subjected to adipogenic induction in the presence of PGE (12.5–50 μg/mL). Oil red O staining demonstrated a dose-dependent reduction in lipid accumulation (Figure 3A). qRT-PCR analysis revealed significant reductions in the expression of key adipogenic transcription factors and markers, including PPARG, C/EBPA, adiponectin, and FABP4, with ~5-fold reductions in PPARG and adiponectin mRNA expression at 50 μg/mL of PGE (Figure 3B). These transcriptional changes were corroborated at the protein level by Western blotting (Figure 3C). The expression of lipogenesis-related genes, FASN, SCD1, SCD2, and ACC, was markedly decreased in a dose-dependent manner, with the most pronounced suppression (~3-fold) observed at the highest PGE concentration (Figure 3D). Collectively, these findings suggest that PGE may attenuate adipogenic and lipogenic pathways in primary preadipocytes, consistent with the observations in 3T3-L1 cells.

### 2.4. PGE Disrupts Transcriptional Regulation and MCE in Early Adipogenesis

**Figure 4:**
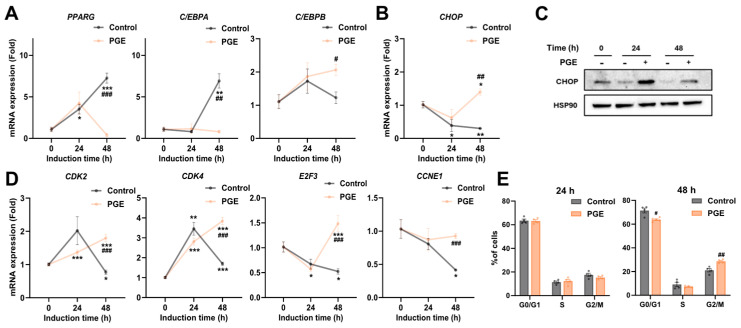
*Primula nutans* Georgi extract (PGE) disrupts transcriptional regulation and mitotic clonal expansion (MCE) during early adipocyte differentiation. (**A**) Time-course qRT-PCR analysis of PPARG, C/EBPA, and C/EBPB expression in 3T3-L1 cells treated with PGE (100 µg/mL) for 24 or 48 h. (**B**,**C**) CHOP mRNA and protein expression were analyzed by qRT-PCR and Western blotting, respectively. (**D**) mRNA expression of MCE-related genes (cyclin E, cyclin D1, Cdk2, Cdk4, and E2F3) was assessed by qRT-PCR. (**E**) The percentage of cells in the G1, S, and G2/M phases was quantified. All data are presented as mean ± SEM (*n* = 4 for qRT-PCR, Western blot, and flow cytometry analyses). Statistical significance: * *p* < 0.05, ** *p* < 0.01, *** *p* < 0.001 vs. control group (0 h); ^#^ *p* < 0.05, ^##^ *p* < 0.01, ^###^ *p* < 0.001 indicates significance compared with the control group (48 h). Each colored dot represents an individual data point.

To investigate the molecular events underlying the early antiadipogenic effects of PGE, time-course analysis was conducted during the initial 48 h of 3T3-L1 differentiation. PGE treatment (100 μg/mL) significantly downregulated the expression of key adipogenic transcription factors, with the PPARG and C/EBPA mRNA levels reduced by approximately 18- and 8-fold, respectively. In contrast, the expression of C/EBPB was modestly increased (~1.5-fold), suggesting a perturbation of transcriptional priming (Figure 4A). The levels of CHOP, an inhibitor of adipogenesis that interferes with C/EBPB function, were markedly upregulated at both the mRNA and protein levels following PGE treatment at 24 and 48 h (Figure 4B,C). These results suggest that CHOP induction may contribute to the inhibitory effect of PGE on adipogenic transcription during early adipogenesis. Moreover, PGE was associated with changes in MCE-related events during early adipogenesis. The expression levels of MCE-associated genes, including CDK2, CDK4, E2F3, and CCNE1, were significantly elevated at 48 h (Figure 4D). Flow cytometry revealed a corresponding increase in the proportion of cells arrested in the G2/M phase, suggesting that PGE may alter cell cycle progression during the later stages of early adipogenesis and may interfere with the proper completion of MCE (Figure 4E and Supplementary Figure S1). Collectively, these findings suggest that PGE may suppress early adipocyte differentiation, potentially associated with changes in adipogenic transcriptional regulators, CHOP induction, and altered cell cycle dynamics during MCE.

### 2.5. PGE Reduces Adiposity and Ameliorates Hepatic Steatosis in HFD-Induced Obese Mice

**Figure 5:**
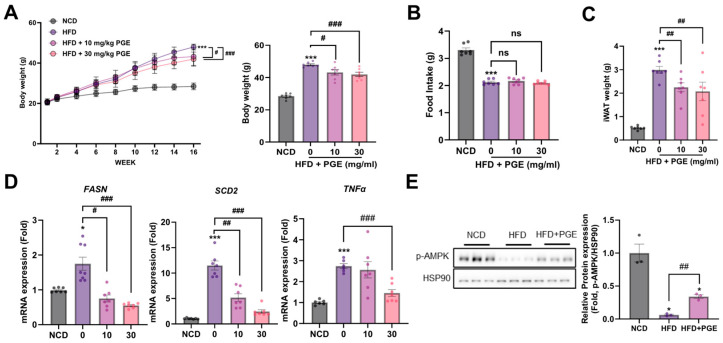
*Primula nutans* Georgi extract (PGE) reduces adiposity and inflammation in HFD-induced obese mice. (**A**) Changes in body weight over the experimental period and final body weight at week 16. (**B**) Food intake monitored throughout the experimental period. (**C**) Inguinal white adipose tissue (iWAT) weight. (**D**) qRT-PCR analysis of FAS and TNFA expression in iWAT. (**E**) Western blot analysis of phosphorylated AMPK (p-AMPK) in iWAT. HSP90 was used as a loading control. Data are presented as the mean ± SEM (*n* = 7 for body weight, food intake, iWAT weight, and qRT-PCR analyses; *n* = 3 for Western blot analysis). * *p* < 0.05, *** *p* < 0.001 vs. normal chow diet (NCD) group; ^#^ *p* < 0.05, ^##^ *p* < 0.01, ^###^ *p* < 0.001 vs. HFD group. Each colored dot represents an individual data point. ns, not significant.

The in vivo antiobesity effects of PGE were evaluated using an HFD-induced obese mouse model. Mice administered PGE (10 or 30 mg/kg) exhibited significantly lower body weight gain than the HFD controls without significant differences in food intake, suggesting that the reduction in adiposity was not attributable to reduced caloric intake (Figure 5A,B and Supplementary Figure S2A). Similarly, the weight of inguinal WAT was significantly decreased in the PGE-treated groups (Figure 5C and Supplementary Figure S2B). Histological analysis of iWAT showed reduced adipocyte size in PGE-treated mice (Supplementary Figure S2C).

At the molecular level, PGE significantly downregulated the expression of FASN, SCD2, and the proinflammatory cytokine tumor necrosis factor-alpha (TNF-α) in iWAT. Notably, TNF-α expression was reduced by approximately 1.5-fold in the high-dose PGE group compared with the HFD controls (Figure 5D). Given that AMPK activation has been associated with antiobesity effects, we assessed AMPK phosphorylation. PGE treatment increased AMPK phosphorylation in adipose tissue (Figure 5E), which may be associated with its antiobesity effects.

**Figure 6:**
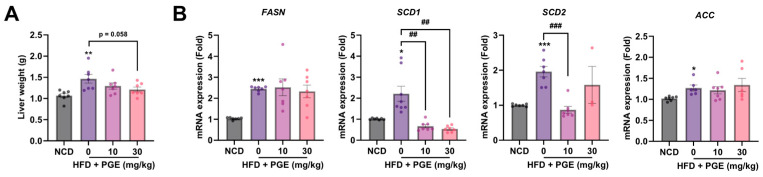
*Primula nutans* Georgi extract (PGE) ameliorates hepatic steatosis and suppresses hepatic lipogenesis. (**A**) Liver weight at sacrifice. (**B**) qRT-PCR analysis of hepatic lipogenic gene expression. Data are presented as the mean ± SEM (*n* = 7). * *p* < 0.05, ** *p* < 0.01, *** *p* < 0.001 vs. NCD group; ^##^ *p* < 0.01, ^###^ *p* < 0.001 vs. HFD group. Each colored dot represents an individual data point.

In the liver, HFD-fed mice showed visibly enlarged livers and substantial lipid deposition (Supplementary Figure S3A). Although liver weights showed a nonsignificant trend toward reduction with PGE treatment (Figure 6A), histological analysis demonstrated clear improvements in hepatic steatosis in the PGE-treated groups (Supplementary Figure S3B). Consistent with these findings, the mRNA expression of hepatic lipogenic genes, including SCD1 and SCD2, which were upregulated by HFD, was markedly reduced following PGE treatment (Figure 6B), suggesting a potential association with reduced hepatic lipid accumulation. Interestingly, the expression levels of FASN and ACC in the liver were nonsignificantly altered by PGE, suggesting that the anti-steatotic effects of PGE may be associated with selective changes in SCD family gene expression rather than broad alterations in hepatic lipogenic gene expression.

### 2.6. PGE Improves Glucose Homeostasis and Insulin Sensitivity in HFD-Fed Mice

**Figure 7:**
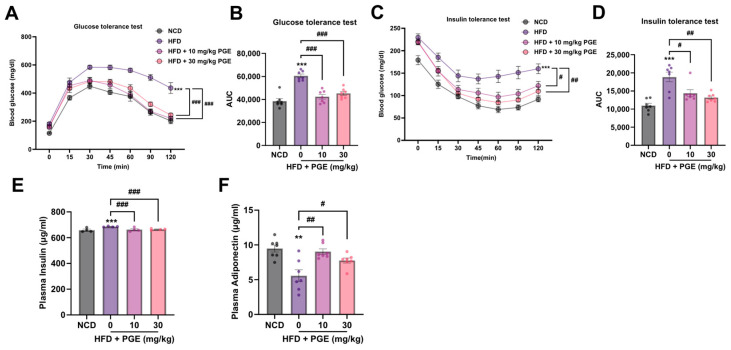
*Primula nutans* Georgi extract (PGE) improves glucose tolerance and insulin sensitivity in HFD-fed mice. (**A**) Glucose tolerance test (GTT): blood glucose levels were measured at 0, 15, 30, 60, 90, and 120 min after glucose injection. (**B**) Area under the curve (AUC) for GTT. (**C**) Insulin tolerance test (ITT): blood glucose levels at indicated time points after insulin injection. (**D**) AUC for ITT. (**E**) Plasma insulin levels measured at week 16. (**F**) Plasma adiponectin levels in each group. All values are presented as mean ± SEM (*n* = 4–7). ** *p* < 0.01, *** *p* < 0.001 vs. NCD group; ^#^ *p* < 0.05, ^##^ *p* < 0.01, ^###^ *p* < 0.001 vs. HFD group. Each colored dot represents an individual data point.

To assess the effects of PGE on glucose metabolism and insulin sensitivity, glucose tolerance tests (GTT) and insulin tolerance tests (ITT) were performed in HFD-induced obese mice. In the GTT, HFD-fed mice exhibited significantly elevated blood glucose levels at all time points compared with the normal chow diet (NCD) controls, indicating impaired glucose tolerance (Figure 7A). Area under the curve (AUC) analysis confirmed this impairment, with AUCs of 60,495 ± 1563.2 for the HFD group and 38,579.5 ± 2161.4 for the NCD group. Notably, PGE treatment was associated with improved glucose clearance in a dose-dependent manner, yielding reduced AUCs of 42,341.2 ± 2031.8 (10 mg/kg) and 45,253.9 ± 2031.8 (30 mg/kg), respectively (Figure 7B). Similarly, ITT results showed delayed glucose clearance in HFD-fed mice compared with the NCD controls, which is indicative of insulin resistance (Figure 7C). The corresponding AUCs were 10,963.9 ± 640.3 for NCD and 18,858.2 ± 1326.7 for HFD mice. PGE administration was associated with improved insulin sensitivity, as reflected by reduced AUCs of 14,379.6 ± 954.2 (10 mg/kg) and 13,172.1 ± 441.1 (30 mg/kg), respectively (Figure 7D).

Plasma insulin levels were slightly elevated in the HFD group compared with the NCD group (685.6 ± 1.9 vs. 659.0 ± 7.5); PGE treatment significantly decreased these levels to 662.2 ± 7.4 (10 mg/kg) and 664.0 ± 3.4 (30 mg/kg), respectively, suggesting improved systemic insulin responsiveness (Figure 7E). Because adiponectin is a key insulin-sensitizing adipokine, its circulating levels were measured. Interestingly, plasma adiponectin concentrations were increased in the PGE-treated groups compared with the HFD group (Figure 7F), suggesting that increased adiponectin levels may contribute to the metabolic effects associated with PGE treatment. Together, these findings suggest that PGE may contribute to improved glucose homeostasis and insulin sensitivity in HFD-induced obese mice, further supporting its potential as a natural candidate for managing insulin resistance and metabolic dysregulation.

### 2.7. Global Natural Products Social Associated Feature-Based Molecular Network-Guided Identification of Metabolites from PGE

**Figure 8:**
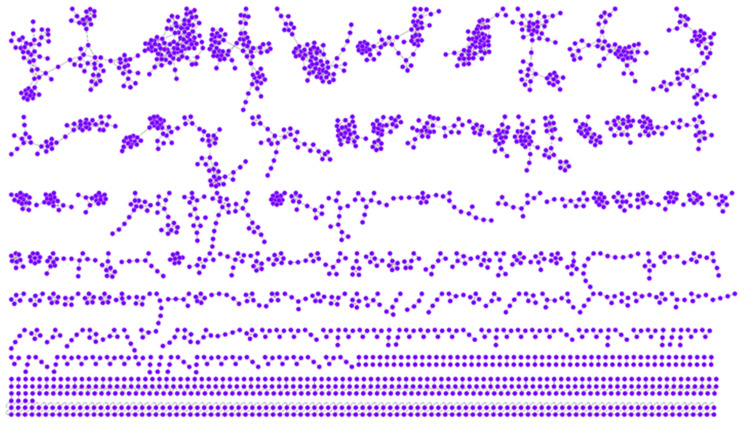
Feature-based molecular network of total *Primula nutans* Georgi extract using positive ion mode.

PGE was analyzed using UHPLC in conjunction with a tandem high-resolution mass spectrometry (MS/MS) detector. The findings indicated that the PGE metabolites displayed mass spectral values between 197 and 1080 Da (Supplementary Figure S4). The mass profile showed high-abundance ion peaks from 290 to 800 Da, encompassing compounds with small-to-medium molecular weights. The chemical profile demonstrated major constituents at 8–11 min with high intensity, indicating that the compounds in this range exhibit high polarity and are likely to possess numerous functional groups or glycoside derivatives. Other abundant peaks were detected at retention intervals of 14.0–16.5 and 19.0–24.5 min (Supplementary Figure S5). This observation suggests that these metabolites have lower polarity or higher molecular weights. To investigate the metabolites from the PGE sample, LC-MS/MS data were analyzed to identify the features of ion peaks and their fragmentation pathway producing fragments. Compound annotation was performed using the MS-Dial and feature-based molecular network workflow available in the Global Natural Products Social Molecular Networking (GNPS) platform (Figure 8). Compounds were tentatively annotated based on spectral similarity between mass features and spectral data obtained from online mass databases (GNPS2, MassBank, and HMDB) [20]. Consequently, 61 putative compounds were annotated from PGE, prominently featuring flavonoids and glycosides (Table 1). Smaller quantities of compounds, such as fatty acids, terpenes, organic acids, and alkaloids, were also tentatively annotated (Supplementary Figure S6). Compounds 13, 14, 17, 20, 22, 24, 45, 46, 53, and 56–59 exhibited relatively high abundance compared with the other signals detected from the chromatograms (Supplementary Figure S7). Based on integrated peak areas, these compounds were considered putative major constituents of PGE.

### 2.8. Identification of Compound and Obesity-Related Targets

**Figure 9:**
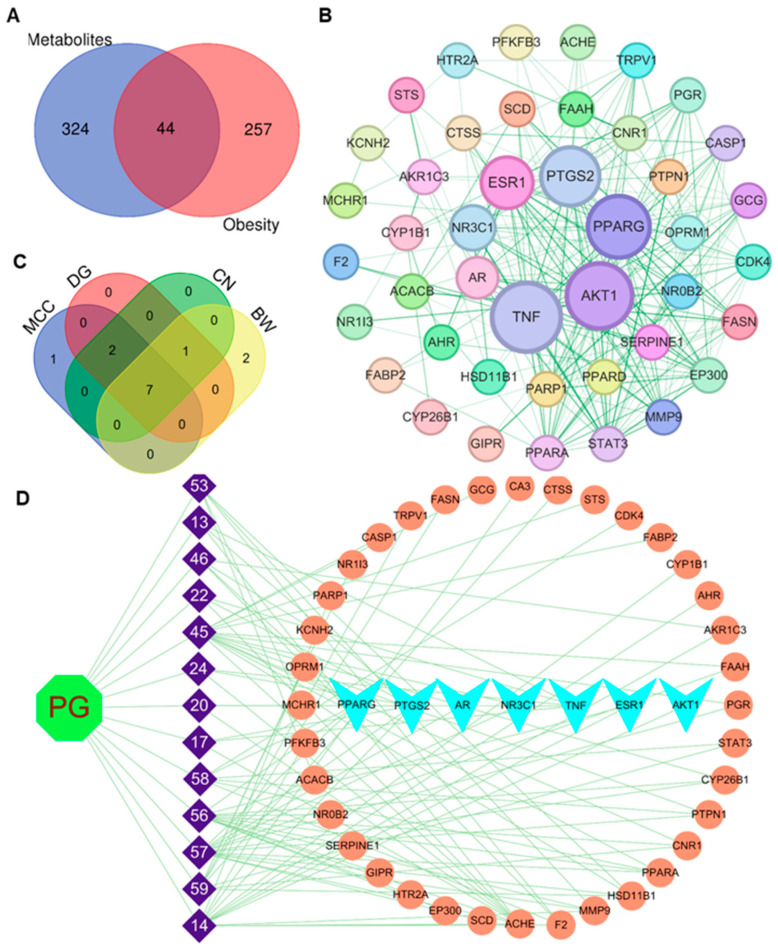
Network pharmacology analysis of *Primula nutans* Georgi extract (PGE)-related antiobesity targets. (**A**) Intersection of potential targets of PGE compounds and known obesity-related genes. (**B**) Protein–protein interaction network of intersected targets; core targets are shown as larger nodes based on degree scores. (**C**) Venn diagram shows the overlap of top-ranked hub genes identified by four algorithms: maximal clique centrality, degree, closeness, and betweenness centrality. (**D**) Compound–target–disease network: blue diamonds represent PGE compounds, cyan V-shapes represent hub genes, and pink ellipses indicate other obesity-related targets.

To identify the molecular targets through which the detected metabolites exert their antiobesity effects, an intersection analysis was conducted between metabolite-related targets and obesity-associated genes. A total of 368 putative targets were predicted for the identified metabolites; 301 obesity-related targets were collected from disease-associated databases. Venn diagram analysis revealed 44 overlapping targets (Figure 9A). These were considered candidate obesity-related targets regulated by metabolites.

### 2.9. Construction of the Protein–Protein Interaction Network

To investigate the functional correlations among the intersecting targets, a protein–protein interaction network was constructed (Figure 9B). The network exhibited a dense and highly interconnected topology, suggesting strong functional coordination among the obesity-related proteins influenced by metabolites. Network topology analysis identified several nodes with high connectivity of seven hub genes, including PPARG, AKT1, TNF, PTGS2, NR3C1, AR, and ESR1, based on centrality scores (maximal clique centrality, degree, closeness, and betweenness), indicating their central roles in the regulatory network.

### 2.10. Gene Ontology Enrichment Analysis

**Figure 10:**
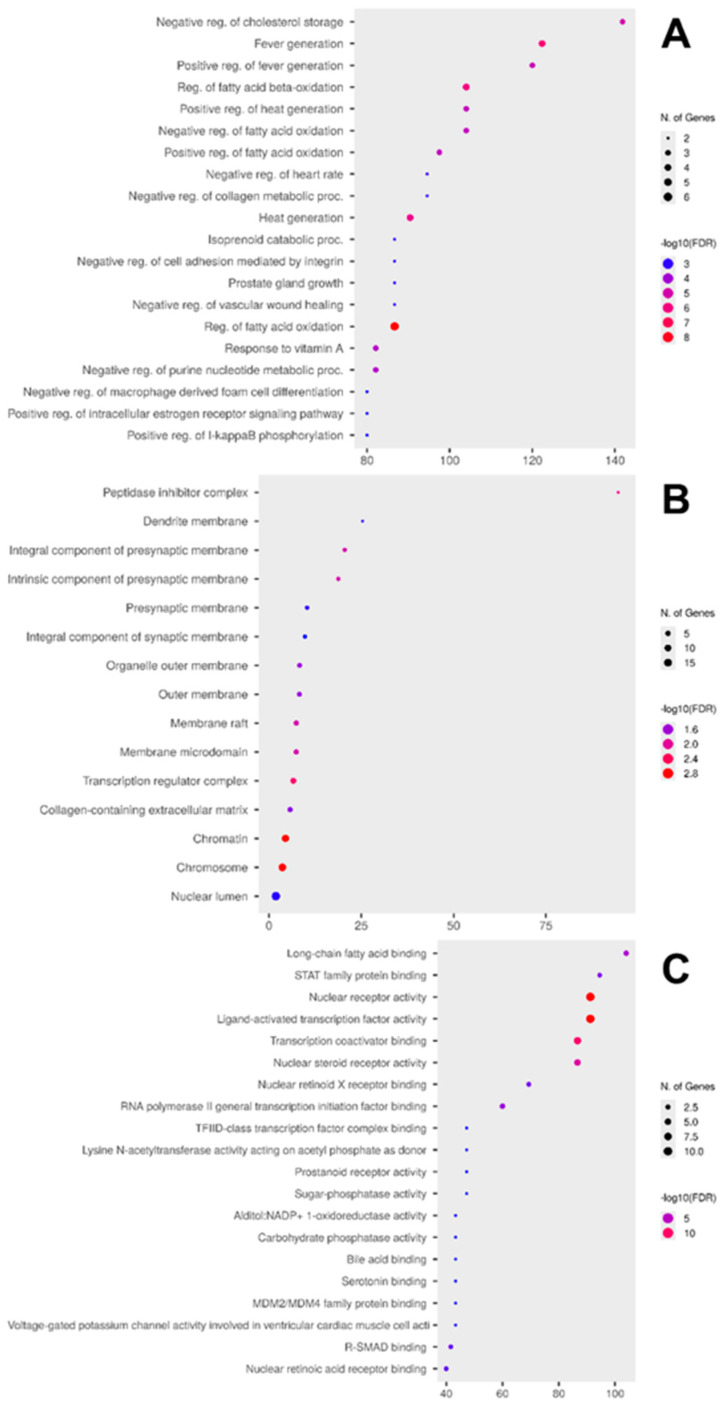
Gene Ontology [12] enrichment analysis of intersected targets. Enrichment terms for (**A**) biological process (BP), (**B**) cellular component (CC), and (**C**) molecular function (MF). Each panel shows the top-ranked GO terms based on adjusted *p*-values and gene counts.

Gene Ontology [12] enrichment analysis revealed 1000 biological processes. The negative regulation of cholesterol storage demonstrated the highest enrichment score among the top 20 ranked biological process terms. This may be associated with the control of lipid accumulation (Figure 10A). Several processes related to fatty acid degradation were significantly enriched, such as the stimulation and suppression of fatty acid oxidation, thereby emphasizing the complex regulation inherent in lipid metabolism.

Processes linked to energy equilibrium, encompassing heat production and its positive regulation, exhibited significant enrichment, suggesting a function in thermogenic processes. Processes pertaining to inflammation, including fever generation and its augmentation, were identified, underscoring the correlation between obesity and immune-metabolic responses. Further enriched biological processes included the inhibition of macrophage-derived foam cell differentiation and the suppression of vascular wound healing, indicating the potential modulation of inflammatory signaling and pathways governed by macrophage activity.

The analysis of GO cellular component enrichment terms revealed that the targets associated with obesity were predominantly found in membrane-related structures, nuclear areas, and complexes involved in transcriptional regulation (Figure 10B). The peptidase inhibitor complex emerged as the most notably enriched cellular component term, accompanied by various membrane-associated elements, such as the presynaptic membrane—an integral component of the synaptic membrane, outer membrane, membrane raft, and membrane microdomain. The results indicate that a significant number of targets play a role in signaling associated with membranes and processes mediated by receptors. Components associated with the nucleus, including chromatin, chromosomes, and nuclear lumen, showed significant enrichment. This, in addition to the transcription regulator complex, points to a significant role for transcription regulation and epigenetic control in the fight against obesity. Enhancement of the collagen-rich extracellular matrix indicates its involvement in the remodeling of the extracellular matrix and regulation of adipose tissue structure.

GO enrichment analysis identified 325 molecular functions, which were mainly linked to lipid binding, nuclear receptor activity, and transcriptional regulation (Figure 10C). The molecular functions that were most significantly enriched were linked to long-chain fatty acid binding, nuclear receptor activity, and ligand-activated transcription factor activity. This finding suggests a direct involvement in lipid sensing and the control of gene expression. The interaction with STAT family proteins, nuclear steroid receptors, and retinoid receptors emphasizes the crucial role of transcription factor-mediated signaling. Various functions related to transcription, including the binding of coactivators, interaction with RNA polymerase II general transcription initiation factors, and association with TFIID-class transcription complexes, were notably enriched, highlighting the importance of regulation at the initiation stage of transcription. Activities of metabolic enzymes, such as aldo-keto reductase, sugar phosphatase, and carbohydrate phosphatase, were identified, indicating further involvement in carbohydrate and lipid processing.

### 2.11. Kyoto Encyclopedia of Genes and Genomes Pathway Enrichment Analysis of Obesity-Related Targets

**Figure 11:**
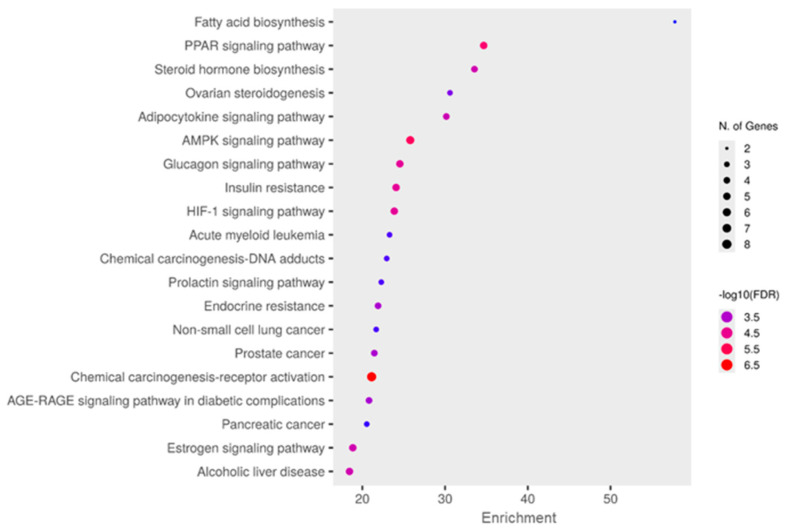
Top 20 KEGG pathway enrichment analyses of *Primula nutans* Georgi extract (PGE)-related antiobesity targets. Bubble plot shows the number of genes per pathway (bubble size) and significance level (color gradient).

Kyoto Encyclopedia of Genes and Genomes (KEGG) pathway enrichment analysis was performed to elucidate the 139 biological pathways associated with obesity-related targets regulated by the identified metabolites. The top 20 significantly enriched pathways are presented in Figure 11, ranked according to statistical significance (−log10 FDR) and gene number.

Pathways primarily associated with lipid metabolism, energy homeostasis, and endocrine regulation were significantly enriched (seen in the enrichment bubble plot, Figure 11). Among these, fatty acid biosynthesis exhibited the highest enrichment ratio, indicating a strong involvement of metabolite-regulated targets in de novo lipid synthesis. The PPAR signaling pathway was highly enriched, with a relatively large number of associated genes and strong statistical significance, highlighting its central role in obesity-related metabolic regulation. Additional metabolism-related pathways, including adipocytokine, glucagon, and insulin resistance, were significantly enriched. These pathways are directly implicated in glucose and lipid metabolism, insulin sensitivity, and energy expenditure, all of which are core pathological features of obesity. Hormone-related pathways, such as steroid hormone biosynthesis, ovarian steroidogenesis, estrogen signaling, and prolactin signaling, were enriched, suggesting that the identified targets influence endocrine balance and hormone-dependent metabolic regulation. Enrichment of the AGE–RAGE signaling pathway in diabetic complications and the HIF-1 signaling pathway indicates a potential link between obesity, chronic inflammation, oxidative stress, and hypoxia-related metabolic dysregulation. Notably, several disease-related pathways, including alcoholic liver disease, chemical carcinogenesis–receptor activation, and endocrine resistance, were identified, reflecting the systemic metabolic and inflammatory consequences of obesity.

### 2.12. Binding Affinity of PGE Components with Proteins Targeting Obesity

**Figure 12:**
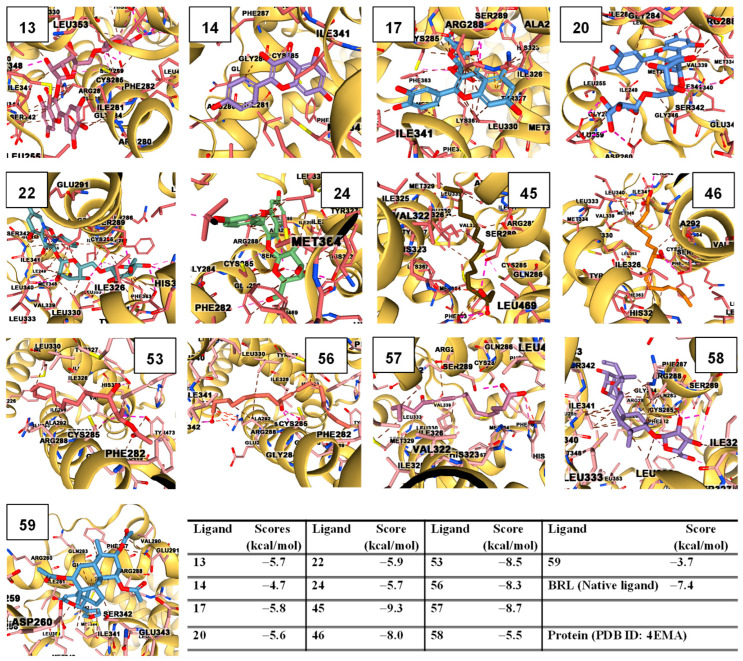
3D interactions of compounds 13, 14, 17, 20, 22, 24, 45, 46, 53, 56, 57, 58, and 59 from *Primula nutans* Georgi extract (PGE) with amino acids when docked onto PPARG (PDB ID: 4EMA).

To assess the antiobesity effects of the major compounds identified from PGE, we performed molecular docking of the major compounds with the target protein. The compounds displayed negative scores of free binding energies with PPARG, C/EPBA, and adiponectin. Briefly, compounds 45, 46, 53, 56, 57, and 58 showed strong binding with low docking scores. The scores were lower than those of the redocked native ligand (BRL, ΔG = −7.4 kcal/mol) with PPARG. Other compounds displayed higher values of free binding energy (Figure 12). Among the compounds docked onto C/EPBA, compound 58 displayed the strongest binding with a docking score of −7.4 kcal/mol; other compounds showed free binding energies ranging from −5.6 kcal/mol to −2.4 kcal/mol. These compounds showed low negative values of free binding energies toward adiponectin. Compounds 13, 17, 20, 22, 45, 46, 53, 58, and 59 demonstrated strong binding with negative values of −5.1, −4.2, −4.1, −5.0, −4.2, −3.9, −4.4, −4.1, and −4.1 kcal/mol, respectively. These findings suggest that these compounds effectively interact with their target proteins. This is due to their interactions with amino acids on the active site of each protein (Supplementary Figures S9 and S10). Therefore, PGE components could be useful in treating diabetes and obesity.

## 3. Discussion

In this study, we investigated the effects of PGE on adipocyte differentiation and metabolic dysfunction using integrated in vitro and in vivo models.

Our results suggest that PGE may inhibit adipocyte differentiation in 3T3-L1 preadipocytes, accompanied by reductions in key adipogenic transcription factors, including PPARG, C/EBPA, adiponectin, and FABP4. Concurrently, PGE reduced the expression of lipogenic enzymes, such as FAS, ACC, and SCD isoforms. These effects were most pronounced when PGE was applied during the early stages of adipogenesis, suggesting that PGE may primarily influence the transcriptional initiation phase of differentiation. Furthermore, consistent anti-adipogenic and anti-lipogenic effects were observed in primary preadipocytes derived from iWAT, confirming the physiological relevance of our findings.

PGE treatment was associated with changes in the expression of early adipogenic regulators. Specifically, PGE treatment was associated with increased CHOP and C/EBPB levels and reduced PPARG and C/EBPA expression. CHOP is known to antagonize adipogenesis by interfering with C/EBPB activity, suggesting that CHOP induction may contribute to the upstream suppression of terminal differentiation markers observed following PGE treatment. Our recent studies have also suggested that CHOP signaling and MCE-associated cell cycle regulation may play important roles during early adipocyte differentiation [21,22]. However, direct causal validation of CHOP-mediated regulation using knockdown or overexpression approaches was not performed in the present study. In addition, PGE altered the expression of several MCE-related genes, such as cyclin E, CDK2, CDK4, and E2F3, with no significant change in cyclin D1 expression. These findings suggest that PGE alters cell cycle progression during the MCE phase and may interfere with the proper completion of early adipogenic events. Because successful adipogenesis requires tightly coordinated cell cycle re-entry and progression during MCE, disruption of these events may impair the transcriptional activation of terminal adipogenic regulators, including PPARG and C/EBPA. In vitro, PGE did not significantly alter AMPK or ERK signaling during early adipocyte differentiation, suggesting that its anti-adipogenic effects may be associated with transcriptional and cell cycle-related mechanisms. However, because AMPK phosphorylation was evaluated only in adipose tissue in vivo, the systemic contribution of AMPK signaling to the metabolic effects of PGE requires further investigation in additional tissues and cellular models.

To validate the in vitro findings, we employed an HFD-induced obese mouse model. Mice treated with PGE (10 or 30 mg/kg) exhibited significantly attenuated body weight gain and reduced iWAT mass without a concomitant reduction in food intake. Histological analysis revealed decreased adipocyte hypertrophy and amelioration of hepatic steatosis in the liver. PGE administration was associated with improved glucose metabolism and insulin sensitivity, as demonstrated by enhanced glucose and insulin tolerance, reduced plasma insulin levels, and elevated adiponectin concentrations. These findings suggest that in obese animals, PGE may contribute to reduced adipocyte lipid accumulation and improved metabolic homeostasis, potentially accompanied by enhanced insulin sensitivity. However, because PGE was administered intraperitoneally in the present study, further investigations using oral administration models will be necessary to determine its pharmacokinetic properties, bioavailability, and translational applicability.

Metabolomic investigations revealed a diverse array of chemical constituents, encompassing flavonoids, fatty acids, glycosides, terpenes, and phenolics. Because flavonoids are known regulators of adipogenesis and lipid metabolism, the flavonoid-rich composition of PGE may contribute to its observed antiobesity effects [23]. Recent studies have further demonstrated that metabolomics-guided analyses of phytochemical-rich extracts can identify bioactive compounds associated with the regulation of adipogenesis, lipid accumulation, and metabolic dysfunction in obesity [24,25]. Flavonoids exert these effects by inhibiting adipogenic transcription factors and lipogenic enzymes, thereby diminishing preadipocyte differentiation and triglyceride accumulation while enhancing fatty acid oxidation and mitochondrial biogenesis [26,27].

Investigations into the efficacy of PGE metabolites in combating obesity, predicated on their principal chemical constituents, revealed 44 overlapping targets shared by the compounds and those associated with obesity. In this set, protein–protein interaction network analysis highlighted hub genes demonstrating extensive interconnectivity among the intersecting targets, indicating their capacity to modulate and influence obesity through diverse biological pathways. Thus, hub genes may exert therapeutic effects through a multitarget mode of action rather than a single molecular pathway. Among them, PPARG is consistent with its established role as a master regulator of adipocyte differentiation, lipid storage, and insulin sensitivity [28]. Modulation of PPARG is a validated strategy in obesity and metabolic syndrome management, and its central position in the network supports its potential importance as a key mediator of metabolite action [29]. AKT1, TNF, ESR1, PTGS2, and other hub genes play pivotal roles in insulin signaling, chronic low-grade inflammation associated with obesity, and glucose metabolism, suggesting potential links between the identified metabolites and pathways involved in metabolic homeostasis [30,31,32]. Recent studies have further highlighted the value of metabolomics-guided network pharmacology approaches for identifying obesity-related molecular targets and elucidating metabolic regulatory pathways involved in adipogenesis and metabolic dysfunction [33]. Functional enrichment and network pharmacology analyses suggested that the identified targets may contribute to the antiobesity effects of PGE-derived metabolites through the regulation of energy expenditure, lipid metabolism, adipogenesis, and glucose homeostasis. Consequently, these findings support the potential involvement of PGE-derived metabolites in the anti-adipogenic effects observed in the in vitro assays. However, further studies are required to isolate individual bioactive compounds and determine their specific contributions to the anti-adipogenic effects of PGE.

Within the molecular function classification, the enrichment of nuclear receptor activity, ligand-activated transcription factor activity, long-chain fatty acid binding, and transcription coactivator interactions highlights the significance of lipid-sensing nuclear receptors and transcriptional regulation in modulating adipogenesis, lipid utilization, and insulin sensitivity. The enriched pathways identified through network pharmacology analyses were consistent with the experimental findings showing reduced adipogenesis, lipid accumulation, and insulin resistance following PGE treatment. Notably, several predicted hub targets, including PPARG, TNF, and AKT1, were closely associated with pathways relevant to adipocyte differentiation, inflammatory signaling, and insulin sensitivity, which were also experimentally modulated by PGE. Molecular docking analyses further suggested favorable interactions between major PGE-derived metabolites and obesity-related targets, including PPARG, C/EBPA, and adiponectin, as indicated by negative binding affinity values. Consequently, these findings support the potential interactions between PGE-derived metabolites and obesity-related targets, consistent with the findings observed in the in vitro assays, although direct experimental validation of these interactions remains necessary. Moreover, because PGE was evaluated as a crude extract, the individual contributions of specific metabolites and the potential synergistic interactions among the constituents remain unclear. Furthermore, the absence of formal blinding procedures and a priori sample size calculations represents a limitation of the present study and should be considered when interpreting the experimental outcomes. Some mechanistic analyses were also performed with relatively small sample sizes, which may limit statistical power and should therefore be interpreted cautiously.

## 4. Materials and Methods

### 4.1. Preparation of PGE

*P. nutans* Georgi was obtained from the International Biological Material Research Center at the Korea Research Institute of Bioscience and Biotechnology (accession number: FBM 278-061). The plant was collected from Tsenkhermandal district, Khentii province, Mongolia, in June 2015. A voucher specimen (KRIB 0070155) is stored at the herbarium of the Korea Research Institute of Bioscience and Biotechnology. In total, 72 g of dried whole plants was powdered; mixed with 1 L of 99.9% (*v*/*v*) methyl alcohol; and extracted through 30 cycles (ultrasonication for 15 min and standing for 120 min per cycle at 40 KHz and 1500 W) at room temperature using an ultrasonic extractor (SDN-900H, Sungdong-Ultrasonic Co., Ltd., Seoul, Republic of Korea). The preparation was filtered (Qualitative Filter No. 100, Hyundai, Micro Co., Ltd., Seoul, Republic of Korea) and dried under reduced pressure to obtain 6.76 g PGE. The extract was lyophilized and dissolved in DMSO for use in subsequent experiments.

### 4.2. Cell Culture and Induction of Adipocyte Differentiation

3T3-L1 preadipocytes were purchased from American Type Culture Collection (Manassas, VA, USA) and maintained in Dulbecco’s modified Eagle’s medium (Welgene Inc., Daegu, Republic of Korea) supplemented with 10% bovine calf serum, 100 U/mL penicillin, and 100 μg/mL streptomycin at 37 °C in a humidified atmosphere with 5% CO_2_. Cells were differentiated by seeding at a density of 5.0 × 10^4^ cells/well in 12-well plates. Two days postconfluence (designated day 0), the cells were treated with differentiation medium containing 10% fetal bovine serum (FBS), 0.5 mM of 3-isobutyl-1-methylxanthine, 1 μM dexamethasone, and 1 μg/mL insulin. On day 2, the medium was replaced with DMEM containing 10% FBS and 0.2 μg/mL insulin, and cells were cultured until day 6, with medium changes every 48 h.

### 4.3. Cell Viability Assay

Cell viability was assessed using the WST-8 Cell Viability Assay Kit (BIOMAX, Seoul, Republic of Korea), according to the manufacturer’s instructions. 3T3-L1 cells were seeded into 96-well plates at a density of 1.0 × 10^4^ cells/well and incubated for 24 h. Cells were treated with varying concentrations of PGE for 24 h and then incubated with WST-8 reagent at 37 °C for 1 h. Absorbance was measured at 450 nm using a microplate reader (iMark, Bio-Rad Laboratories, Hercules, CA, USA); cell viability was calculated relative to the untreated controls (set at 100%).

### 4.4. Oil Red O Staining

Differentiated 3T3-L1 cells were washed twice with Dulbecco’s phosphate-buffered saline; fixed with 3.7% formalin for 10 min; dehydrated two times with 100% propylene glycol for 30 min at 30 rpm; and stained with 0.7% Oil red O solution for 15 min. Excess stain was removed by washing three times with distilled water, and stained lipid droplets were visualized using light microscopy at 200× magnification.

### 4.5. Quantitative Real-Time PCR

Total RNA was extracted from cells or tissues using RiboEx reagent (GeneAll Biotechnology, Seoul, Republic of Korea) and reverse-transcribed to cDNA using the ReverTra Ace qPCR RT Kit (Toyobo Co., Ltd., Osaka, Japan). Quantitative PCR was performed with SFCgreen PCR Master Mix (BIOFACT, Daejeon, Republic of Korea) using the CFX Connect Real-Time PCR System (Bio-Rad Laboratories, Hercules, CA, USA). Relative mRNA ex-pression was normalized to 36B4 as a housekeeping gene. Most of the primer sequences used in the experiment are listed in our previous studies [17,34,35].

### 4.6. Western Blot Analysis

Cells were lysed in a mammalian protein extraction reagent (Thermo Fisher Scientific, Waltham, MA, USA) supplemented with protease and phosphatase inhibitors (GenDEPOT, Baker, TX, USA). Lysates were centrifuged at 12,000 rpm for 10 min at 4 °C, and protein concentrations were determined using a BCA assay kit. Equal amounts of protein were separated by SDS-PAGE and transferred to PVDF membranes. The membranes were blocked in 5% bovine serum albumin in TBST for 1 h and incubated overnight at 4 °C with the following primary antibodies: PPARG (Cat. No. 2443, Cell Signaling Technology, Danvers, MA, USA), adiponectin (Cat. No. MA1-054, Thermo Fisher Scientific, Waltham, MA, USA), CHOP (Cat. No. 2895, Cell Signaling Technology, Danvers, MA, USA), and HSP90 (Cat. No. SC-13119, Santa Cruz Biotechnology, Dallas, TX, USA). The membranes were washed and incubated with HRP-conjugated secondary antibodies (1:4000; Bio-Rad Laboratories, Hercules, CA, USA). Protein bands were visualized using an ECL detection system and imaged using the iBright CL1500 system (Thermo Fisher Scientific, Waltham, MA, USA).

### 4.7. Isolation and Differentiation of Stromal Vascular Cells (SVCs)

Primary SVCs were isolated from the iWAT of male C57BL/6 mice. Adipose tissue was minced and digested with 2 mg/mL collagenase D in Hank’s Balanced Salt Solution at 37 °C for 40 min with shaking (160 rpm), as described previously [34]. The digested tissue was filtered through a 100-μm strainer and centrifuged at 1500 rpm for 5 min. The cell pellet was resuspended in red blood cell lysis buffer (Thermo Fisher Scientific, Waltham, MA, USA) and filtered through a 40-μm strainer. For adipogenic induction, cells were cultured for 2 d and then treated with differentiation medium containing 0.2 mM indomethacin, 1 μM dexamethasone, 0.5 mM 3-isobutyl-1-methylxanthine, 1 μg/mL insulin, and 1 μM rosiglitazone in DMEM supplemented with 10% FBS. From day 2 to day 6, the medium was replaced every 48 h with DMEM containing 10% FBS, insulin (1 μg/mL), and rosiglitazone (1 μM). All procedures were approved by the Institutional Animal Care and Use Committee of Mokpo National University (Approval No. MNU-IACUC-2023-006).

### 4.8. Flow Cytometry Assay

To evaluate changes in the phases of the cell cycle, 3T3-L1 cells were dissociated using Trypsin-EDTA solution (Welgene, Daegu, Republic of Korea), washed with Dulbecco’s phosphate-buffered saline, and fixed in 70% ethanol at −20 °C overnight. Fixed cells were stained with 2 μg/mL propidium iodide (Cat. No. P3566, Thermo Fisher Scientific, Waltham, MA, USA), washed twice with Dulbecco’s phosphate-buffered saline, and treated with RNase A (100 μg/mL) (Cat. No. PE290-25h, BIOFACT, Daejeon, Republic of Korea). Propidium iodide fluorescence intensity and cell cycle distribution were analyzed using a CytoFLEX flow cytometer (Beckman Coulter, Brea, CA, USA) with CytExpert software (Version 2.6.0.105, Beckman Coulter, Brea, CA, USA). Results are presented as content histograms for each dataset.

### 4.9. Animal Study and PGE Administration

Male C57BL/6 mice (6 weeks old) were housed under specific pathogen-free conditions with a 12-h light/dark cycle and acclimatized for 1 week. The mice were fed an HFD for 16 weeks and randomly assigned to four groups (*n* = 7 per group): NCD, HFD control, HFD + PGE 10 mg/kg, and HFD + PGE 30 mg/kg. For PGE administration, PGE powder was dissolved in DMSO and diluted in 30% hydroxypropyl-β-cyclodextrin in PBS to prepare final concentrations of 3 and 1 mg/mL. The solution was sonicated for 5 min and vortexed for 1 min three times to ensure homogeneity. Mice received intraperitoneal injections of PGE at doses of 10 or 30 mg/kg, three times per week, throughout the study. Body weight and food intake were recorded regularly. At the end of the experimental period, mice were fasted overnight and euthanized. Mice blood and tissue samples were collected for analysis. Plasma insulin levels were measured using a commercial ELISA kit (Cat. No. E-EL-M1382, Elabscience, Houston, TX, USA). Plasma adiponectin levels were measured using a commercial ELISA kit (Cat. No. MRP300, R&D systems, Minneapolis, MN, USA). All experimental procedures were approved by the Institutional Animal Care and Use Committee of Mokpo National University (Approval No. MNU-IACUC-2023-005).

### 4.10. Glucose Tolerance and Insulin Tolerance Tests

At week 16, GTT and ITT were conducted according to standard procedures. For GTT, mice were fasted for 16 h and injected intraperitoneally with 1.5 mg/g glucose (in PBS). Blood glucose levels were measured from tail vein samples at 0, 15, 30, 45, 60, 90, and 120 min. ITT was conducted after 3 d after 6-h fasting by administering 1 U/g insulin (in PBS) and measuring glucose at the same time intervals.

### 4.11. Histological Analysis

Mice were euthanized after overnight fasting. Mice tissues (liver, iWAT, and BAT) were collected and fixed in 10% buffered formalin. Hematoxylin and eosin staining was performed by KP&T Technology (Cheongju, Republic of Korea), and stained sections were evaluated under a microscope for histological analysis.

### 4.12. Analytical Procedures of Metabolites from PGE

The PGE extract was dissolved in high grade MeOH before filtering through a 0.22 μm PTFE membrane filter (Agilent Technologies, Santa Clara, CA, USA) prior to analysis. The extract solution was then analyzed by using a Vanquish UHPLC associated with an Orbitrap Exploris 120 mass spectrometer (Thermo Fisher Scientific, Waltham, MA, USA) [35]. The stational phase was a Waters Acquity UPLC HSS T3 column (size: 4.6 × 100 mm, particle size: 1.8 μm, Waters, Milford, MA, USA) maintained at 40 °C. The flow rate was set at 0.2 mL/min along with an injection volume of 4 μL. The mobile phase consisted of solvent A (0.1% formic acid in water) and solvent B (0.1% formic acid in acetonitrile) buffering with 0.01% ammonia applied as a gradient solvent system: 8–15% B (0–4 min), 15–32% B (4–8 min), 32–53% B (8–11 min), 53–100% B (11–24 min), held for 3 min. The system is equivalent to initial conditions at 8% B for 1 min.

### 4.13. Annotation of Metabolites Identified from PGE

The raw data file was converted using MZmine version 3.9.0 and uploaded to the GNPS platform (University of California, San Diego, La Jolla, CA, USA) [20] repository with task ID_ 7b470cfee5f64010bf83483f05be94ec. The compounds were annotated using MS-DIAL 5 software (Riken Center for Sustainable Resource Science, Yokohama City, Kanagawa, Japan). The output data were analyzed for isotope patterns and mass duplication to spectral-matching scores and features between experimental and reference data against multiple online mass spectral databases [36]. A molecular network was obtained from GNPS-FBMN, visualized using the Cytoscape (Version 3.10.1, Institute for Systems Biology, Seattle, WA, USA) program. Displayed nodes represent molecular attributions, and edges display similar structure features based on clusters of specific compounds. The cosine score factor was applied to retain structurally relevant connections and visualization.

### 4.14. Definition of Compounds and Obesity-Related Targets and Protein–Protein Interaction Network

SMILES of the selected compounds were obtained from the PubChem database (https://pubchem.ncbi.nlm.nih.gov/, accessed on 15 November 2025). SMILES were inputted into the SwissTargetPrediction webserver (https://www.swisstargetprediction.ch/predict.php, accessed on 20 November 2025). Obesity-related targets were retrieved from the DisGeNet website (https://disgenet.com/, accessed on 29 November 2025). All targets were filtered by selecting Homo Sapiens as the species and a threshold cutoff of >0 [37]. A Venn diagram was employed to determine the intersection between compound and disease targets through an online platform (https://bioinfogp.cnb.csic.es/tools/venny/, accessed on 2 December 2025). Overlapped targets were inserted into the STRING database (https://string-db.org/, accessed on 2 December 2025) by selecting H. Sapiens as the species and a confidence score of >0.7. The generated data was visualized using Cytoscape (Version 3.10.1). Hub genes were defined due to high values of closeness, betweenness, maximal clique centrality, and degree scores using the cytoHubba plugin in Cytoscape software.

### 4.15. GO and KEGG Enrichment Analysis

KEGG and GO (biological process, MF, and cellular component) enrichment terms were defined in the ShinyGO 0.82 (http://bioinformatics.sdstate.edu/go/ accessed on 5 December 2025) database by setting cutoff values of *p* < 0.05 and FDR < 0.01. The KEGG mapper (https://www.genome.jp/kegg/, accessed on 5 December 2025) highlighted the top pathway and demonstrated its distinct molecular mechanism within the pathway.

### 4.16. Prediction of Binding Affinity Using Molecular Docking

To predict the binding affinity of major compounds identified from PGE, we performed molecular docking procedures using the Hyperlab web platform [38]. The proteins were defined from the RCSB website (https://www.rcsb.org/, accessed on 11 December 2025) regarding PPARG (PDB ID: 4EMA), C/EPBA (PDB ID: 1NWWQ), and adiponectin (PDB ID: 6KS0) [39]. The sdf formats of ligands were collected through the PubChem website (https://pubchem.ncbi.nlm.nih.gov/, accessed on 15 July 2025). The PDB ID of each protein along with the ligand was inserted into the Hyperlab web server (https://hyperlab.ai/en/, accessed on 9, 12 and 13 August 2025). Docking was performed automatically by applying the default setting. The output data were analyzed and visualized using a web platform.

### 4.17. Statistical Analysis

All data are presented as means ± standard error of the mean (SEM). Statistical analyses were performed using GraphPad Prism 10.5.0 (GraphPad Software, San Diego, CA, USA). Comparisons between two groups were conducted using an unpaired two-tailed Student’s *t*-test or Welch’s *t*-test, as appropriate, while multiple-group comparisons were analyzed using one-way ANOVA followed by appropriate post hoc tests. A *p*-value of < 0.05 was considered statistically significant.

No formal a priori sample size calculation was performed in this study. Animals were allocated to experimental groups based on standard practices commonly used in exploratory animal studies. In addition, blinding was not applied during animal allocation, outcome assessment, or data analysis.

## 5. Conclusions

This study suggests that PGE may exert antiobesity and insulin-sensitizing effects associated with the regulation of adipogenesis and lipogenesis, maintenance of glucose homeostasis, and modulation of metabolic pathways. Although 61 metabolites were identified in PGE, the specific compounds primarily responsible for the anti-adipogenic effects were not experimentally isolated or validated in the present study. The combined use of in vitro, in vivo, and in silico approaches supports the potential of PGE as a natural candidate for managing obesity and associated metabolic disorders. In addition, because PGE was administered intraperitoneally in the present study, further studies using oral administration models, as well as evaluations of pharmacokinetic properties, long-term safety, and toxicity profiles, will be necessary prior to clinical application. Future studies are warranted to isolate individual active compounds, assess their pharmacokinetics and bioavailability, and further elucidate their molecular mechanisms of action.

## Figures and Tables

**Table 1:** Identification of metabolites from PGE using positive ion mode.

No.	Compound	RT (min)	*m*/*z* (Da)	Formula	Adduct	Error (ppm)	Class
1	1-*β*-D-glucopyranosyl-L-tryptophan	5.4968	367.1477	C_17_H_22_N_2_O_7_	[M + H]^+^	0.006	Glycosides
2	(*E*)-3-[4-[(2*S*,3*R*,4*S*,5*S*,6*R*)-3,4,5-Trihydroxy-6-(hydroxymethyl)oxan-2-yl]oxyphenyl]prop-2-enoic acid	5.7045	344.1319	C_15_H_18_O_8_	[M + NH_4_]^+^	0.006	Glycosides
3	Thymol-*β*-D-glucoside	6.233	313.1601	C_16_H_24_O_6_	[M + H]^+^	2.229	Glycosides
4	1-(4-Hydroxyphenyl)-3-[(2*R*,3*R*,4*S*,5*S*,6*R*)-3,4,5-trihydroxy-6-(hydroxymethyl)oxan-2-yl]oxypropan-1-one	6.233	346.1474	C_15_H_20_O_8_	[M + NH_4_]^+^	2.347	Glycosides
5	(*Z*)-3-[4-Methoxy-2-[(2*S*,3*R*,4*S*,5*S*,6*R*)-3,4,5-trihydroxy-6-(hydroxymethyl)oxan-2-yl]oxyphenyl]prop-2-enoic acid	6.277	374.1422	C_16_H_20_O_9_	[M + NH_4_]^+^	2.198	Glycosides
6	Aloesin	6.451	395.1287	C_19_H_22_O_9_	[M + H]^+^	2.588	Glycosides
7	(2*R*,3*S*,4*S*,5*R*,6*R*)-2-(Hydroxymethyl)-6-[[(2*R*,3*S*,4*S*,5*R*,6*R*)-3,4,5-trihydroxy-6-phenylmethoxyoxan-2-yl]methoxy]oxane-3,4,5-triol	6.591	450.194	C_19_H_28_O_11_	[M + NH_4_]^+^	2.508	Glycosides
8	Epsilon-viniferin	6.591	455.1496	C_28_H_22_O_6_	[M + H]^+^	1.4742	Phenols
9	Unknown	6.709	935.2595	C_28_H_34_N_22_O_16_	[M + H]^+^	0.073	Unknown
10	Unknown	6.709	957.242	C_44_H_40_N_6_O_19_	[M + H]^+^	0.036	Unknown
11	Unknown	7.996	811.1853	C_39_H_26_N_10_O_11_	[M + H]^+^	0.203	Unknown
12	Rutin^+^	8.286	611.1565	C_27_H_30_O_16_	[M + H]^+^	2.79	Flavonoids
13	Myricetin-3-rutinoside	8.604	627.1516	C_27_H_30_O_17_	[M + H]^+^	0.012	Flavonoids
14	Kaempferol^+^	8.721	287.0529	C_15_H_10_O_6_	[M + H]^+^	0.016	Flavonoids
15	Kaempferol-3-*O*-galactoside-7-*O*-rhamnoside	8.913	595.1618	C_27_H_30_O_15_	[M + H]^+^	1.665	Flavonoids
16	Unknown	9.931	498.2567	C_23_H_31_N_9_O_4_	[M + H]^+^	0.929	Unknown
17	Hirsutrin	9.962	465.0999	C_19_H_28_O_11_	[M + H]^+^	2.082	Flavonoids
18	Unknown	10.246	558.1783	C_33_H_19_N_9_O	[M + H]^+^	0.337	Unknown
19	Isorhamnetin	10.476	317.0636	C_16_H_12_O_7_	[M + H]^+^	2.257	Flavonoids
20	Isorhamnetin-3-*O*-rutinoside	10.474	625.1725	C_28_H_32_O_16_	[M + H]^+^	2.053	Flavonoids
21	Kaempferol 3-(2″,4″-di-(*E*)-p-coumaroylrhamnoside)	10.517	725.1856	C_39_H_32_O_14_	[M + H]^+^	0.505	Flavonoids
22	5,7-Dihydroxy-2-(4-hydroxy-3-methoxyphenyl)-3-[(2*S*,3*R*,4*S*,5*R*,6*R*)-3,4,5-trihydroxy-6-[[(2*R*,3*R*,4*R*,5*R*,6*S*)-3,4,5-trihydroxy-6-methyloxan-2-yl]oxymethyl]oxan-2-yl]oxychromen-4-one	10.742	197.116	C_11_H_16_O_3_	[M + H]^+^	0.005	Flavonoids
23	Unknown	10.732	653.1677	C_38_H_20_N_8_O_4_	[M + H]^+^	0.464	Unknown
24	Astragalin^+^	10.863	449.1052	C_21_H_20_O_11_	[M + H]^+^	0.006	Flavonoids
25	[(2*S*,3*S*,4*R*,5*R*)-4-Hydroxy-2,5-bis(hydroxymethyl)-2-[(2*R*,3*R*,4*S*,5*S*,6*R*)-3,4,5-trihydroxy-6-(hydroxymethyl)oxan-2-yl]oxyoxolan-3-yl] (E)-3-(4-hydroxy-3-methoxyphenyl)prop-2-enoate	11.168	541.1521	C_22_H_30_O_14_	[M + Na]^+^	1.127	Organic acids
26	Unknown	11.168	558.1785	C_20_H_27_N_7_O_12_	[M + H]^+^	0.819	Unknown
27	Luteolin-4′-*O*-glucoside	11.648	449.1052	C_21_H_20_O_11_	[M + H]^+^	0.013	Flavonoids
28	Unknown	11.692	464.1886	C_19_H_25_N_7_O_7_	[M + H]^+^	0.285	Unknown
29	9-Methoxy-7-[4-[3,4,5-trihydroxy-6-[[3,4,5-trihydroxy-6-(hydroxymethyl)oxan-2-yl]oxymethyl]oxan-2-yl]oxyphenyl]-[1,3]dioxolo [4,5-g]chromen-8-one	11.715	659.1549	C_29_H_32_O_16_	[M + Na]^+^	2.408	Flavonoids
30	Unknown	11.75	764.3082	C_30_H_37_N_17_O_8_	[M + H]^+^	0.016	Unknown
31	Unknown	11.761	794.3186	C_31_H_39_N_17_O_9_	[M + H]^+^	0.427	Unknown
32	Iridin	11.983	523.1417	C_24_H_26_O_13_	[M + H]^+^	2.101	Flavonoids
33	Unknown	11.948	764.3081	C_32_H_49_N_3_O_18_	[M + H]^+^	0.29	Unknown
34	Unknown	12.215	632.2566	C_14_H_33_N_17_O_12_	[M + H]^+^	0.131	Unknown
35	Unknown	12.418	1066.5733	C_58_H_83_NO_17_	[M + H]^+^	0.012	Unknown
36	2-[4-Hydroxy-2-(hydroxymethyl)-6-[[6-hydroxy-7,9,13-trimethyl-6-[3-methyl-4-[3,4,5-trihydroxy-6-(hydroxymethyl)oxan-2-yl]oxybutyl]-5-oxapentacyclo [10.8.0.02,9.04,8.013,18]icos-18-en-16-yl]oxy]-5-(3,4,5-trihydroxy-6-methyloxan-2-yl)oxyoxan-3-yl]oxy-6-methyloxane-3,4,5-triol	12.418	1071.5285	C_51_H_84_O_22_	[M + Na]^+^	1.367	Phenols
37	Matsutakeside I	12.92	638.244	C_30_H_36_O_14_	[M + NH_4_]^+^	0	Phenols
38	Enoxolone	13.337	471.3438	C_30_H_46_O_4_	[M + H]^+^	2.108	Terpenes
39	Scrophularoside A8	13.337	786.2826	C_35_H_44_O_19_	[M + NH_4_]^+^	2.096	Terpenes
40	11-Hydroxy-9-(hydroxymethyl)-2-methoxycarbonyl-2,6a,6b,9,12a-pentamethyl-10-(3,4,5-trihydroxyoxan-2-yl)oxy-1,3,4,5,6,6a,7,8,8a,10,11,12,13,14b-tetradecahydropicene-4a-carboxylic acid	13.689	682.4137	C_36_H_56_O_11_	[M + NH_4_]^+^	2.147	Terpenes
41	Unknown	13.752	1080.5887	C_59_H_85_NO_17_	[M + H]^+^	0.261	Unknown
42	Unknown	14.491	536.1731	C_21_H_25_N_7_O_10_	[M + H]^+^	0.764	Unknown
43	Unknown	14.523	1077.5419	C_44_H_76_N_12_O_19_	[M + H]^+^	0.234	Unknown
44	Unknown	15.017	644.293	C_16_H_37_N_17_O_11_	[M + H]^+^	0.047	Unknown
45	9-OxoOTrE	15.4	275.1989	C_18_H_28_O_3_	[M-H_2_O + H]^+^	0.004	Fatty acids
46	9(S)-HpOTrE	15.4006	293.2093	C_18_H_30_O_4_	[M-H_2_O + H]^+^	0.006	Fatty acids
47	Unknown	15.718	494.1627	C_19_H_23_N_7_O_9_	[M + H]^+^	0.447	Unknown
48	Unknown	15.663	678.4534	C_33_H_63_N_3_O_11_	[M + H]^+^	2.081	Unknown
49	Unknown	15.718	494.1627	C_19_H_23_N_7_O_9_	[M + H]^+^	0.447	Unknown
50	9,12,13-Trihydroxy-15-octadecenoic acid	18.21	348.2724	C_18_H_34_O_5_	[M + NH_4_]^+^	1.14	Fatty acids
51	Unknown	19.665	728.4022	C_28_H_61_N_3_O_18_	[M + H]^+^	0.008	Unknown
52	(2*S*,3*S*,4*R*)-2-Aminooctadecane-1,3,4-triol	20.976	318.2984	C_18_H_39_NO_3_	[M + H]^+^	3.069	Alkaloids
53	1,4-Dihydroxyheptadec-16-en-2-yl acetate	21.426	351.2508	C_19_H_36_O_4_	[M + Na]^+^	0.521	Organic acids
54	Unknown	21.765	470.3811	C_23_H_47_N_7_O_3_	[M + H]^+^	0.384	Unknown
55	Unknown	22.138	393.2589	C_18_H_36_N_2_O_7_	[M + H]^+^	0.586	Unknown
56	9*S*-Hydroxy-10*E*,12*Z*,15*Z*-octadecatrienoic acid	22.318	277.1783	C_18_H_30_O_3_	[M-H_2_O+H]^+^	0.136	Fatty acids
57	13-Keto-9*Z*,11*E*-octadecadienoic acid	23.087	295.2251	C_18_H_30_O_3_	[M-H_2_O + H]^+^	0.006	Fatty acids
58	(2*R*,3*R*,4*S*,5*R*,6*R*)-2-[[7-[(2*R*,3*R*,4*R*,5*S*)-3,4-Dihydroxy-5-(hydroxymethyl)oxolan-2-yl]oxy-2-ethenyl-2,4b,8,8-tetramethyl-4,4a,5,6,7,8a,9,10-octahydro-3H-phenanthren-3-yl]oxy]-6-methyloxane-3,4,5-triol	23.958	621.3029	C_31_H_50_O_10_	[M + K]^+^	0.982	Phenols
59	Deoxykhivorin	25.933	593.2719	C_32_H_42_O_9_	[M + Na]^+^	2.161	Organic acids
60	12-O-[octa-2*Z*,4*E*-dienoyl]-13-isobutyroyloxy-4-deoxyphorbol	26.145	563.2982	C_32_H_44_O_7_	[M + Na]^+^	0.433	Terpenes
61	Phaeophorbide A	26.441	593.2719	C_35_H_36_N_4_O_5_	[M + H]^+^	0.004	Alkaloids

^+^ Reference standard.

## Data Availability

The original contributions presented in this study are included in the article/Supplementary Materials. Further inquiries can be directed to the corresponding author.

## Supplementary Materials

The following supporting information can be downloaded at: https://www.mdpi.com/article/10.3390/ijms27114693/s1.

## Abbreviations

The following abbreviations are used in this manuscript:

PGE	*P. nutans* Georgi extract
PPARG	Peroxisome proliferator-activated receptor gamma
C/EBPA	CCAAT/enhancer-binding protein alpha
MCE	Mitotic clonal expansion
CHOP	C/EBP homologous protein
FASN	Fatty acid synthase
SCD1	Stearoyl-CoA desaturase 1
iWAT	Inguinal white adipose tissue
GTT	Glucose tolerance tests
ITT	Insulin tolerance tests
KEGG	Kyoto Encyclopedia of Genes and Genomes
