# Supplementary material for: Primula nutans Georgi Extract Inhibits Early Adipogenesis Through CHOP-Associated Regulation and Ameliorates Obesity and Insulin Resistance
Source: Int J Mol Sci. 2026 May 22;27(11):4693. doi: 10.3390/ijms27114693 (PMC13257237; doi:10.3390/ijms27114693)
Supplement: Supplementary file 1 [file ijms-27-04693-s001.zip › ijms-4307053-supplementary.pdf]

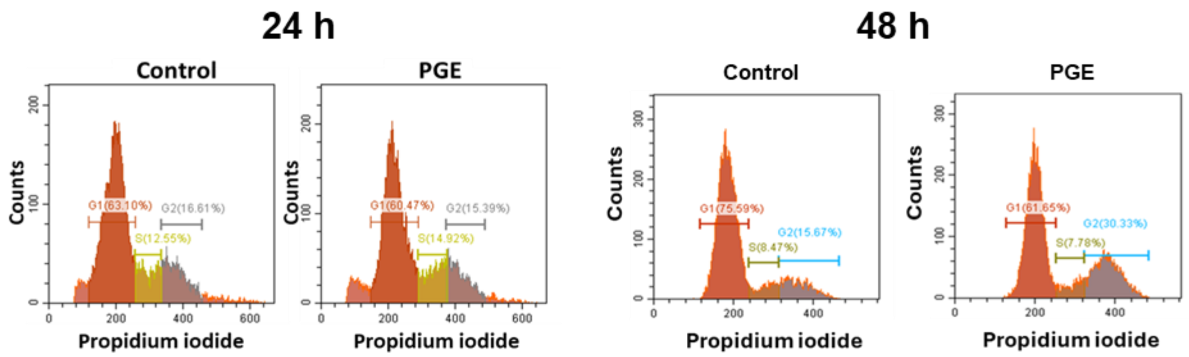

Figure S1. *Primula nutans* Georgi extract (PGE) alters cell cycle progression during early adipogenesis. 3T3-L1 cells were treated with PGE (100 µg/mL) for 24 or 48 h, and cell cycle distribution was analyzed by flow cytometry using propidium iodide staining. Representative flow cytometric profiles of cell cycle distribution are shown.

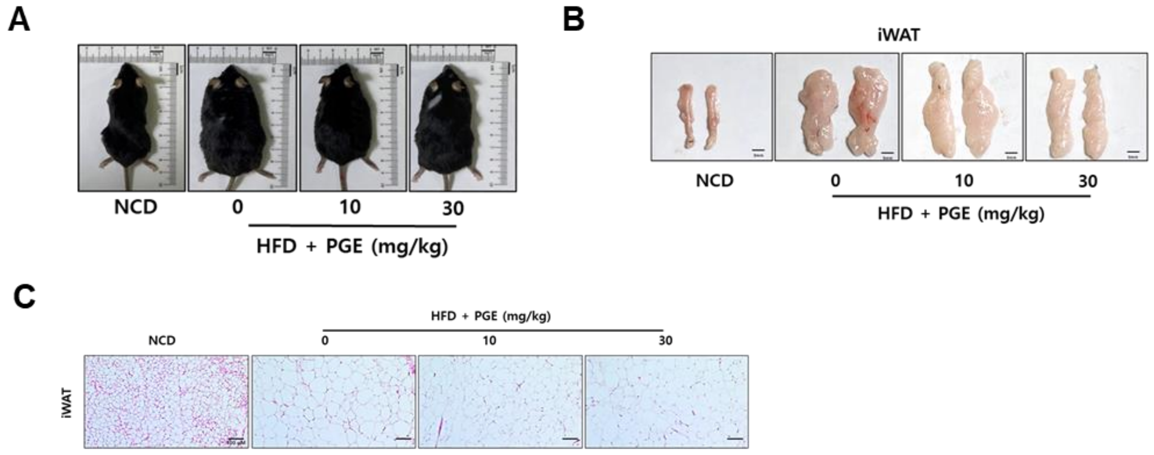

Figure S2. *Primula nutans* Georgi extract (PGE) reduces adiposity in HFD-induced obese mice. (**A**) Representative images of mice after 16 weeks of dietary intervention. (**B**) Representative images of inguinal white adipose tissue (iWAT) from each experimental group. (**C**) Representative hematoxylin and eosin (H&E) staining of iWAT sections. Scale bar = 100 µm.

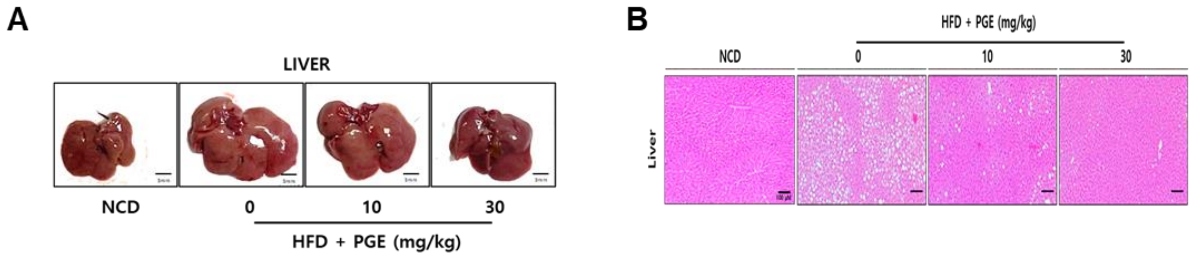

Figure S3. *Primula nutans* Georgi extract (PGE) ameliorates hepatic steatosis in HFD-induced obese mice. (**A**) Representative liver images from each experimental group at the end of the experimental period. (**B**) Representative hematoxylin and eosin (H&E) staining of liver sections. Scale bar = 100 μm.

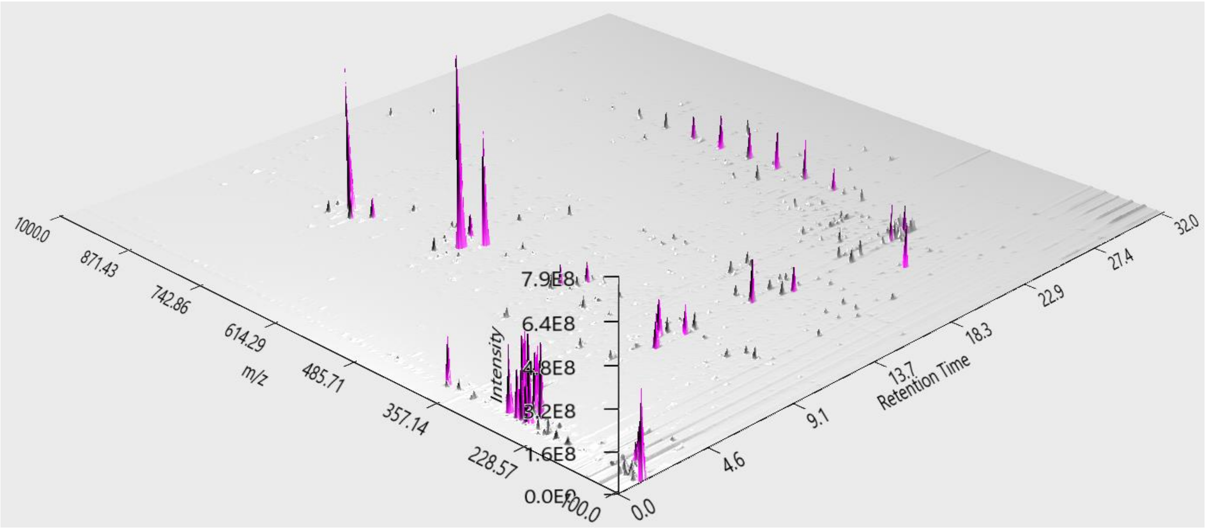

Figure S4. 3D chromatograms of total extract from PGE sample detected in the positive ion mode.

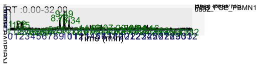

Figure S5. 2D chromatograms of total extract from PGE sample detected in the positive ion mode.

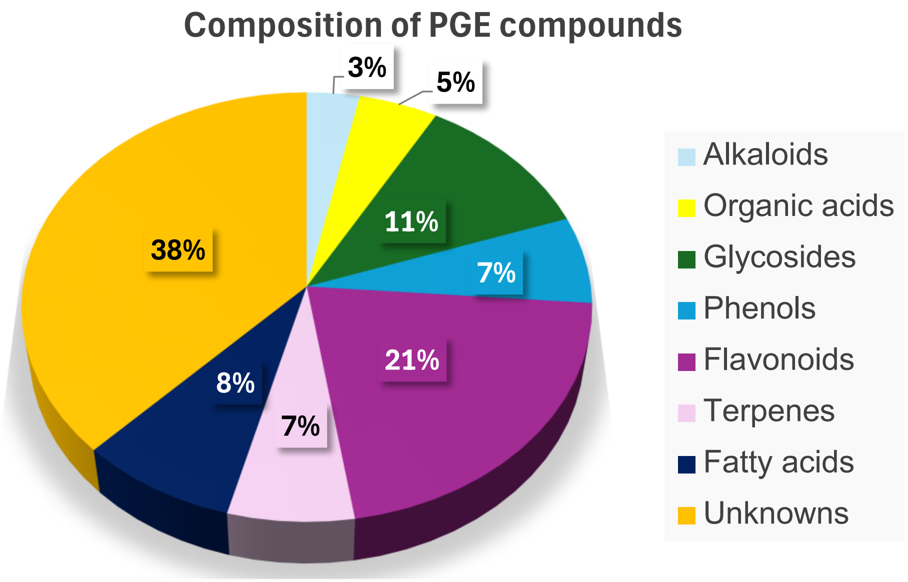

Figure S6. Chemical classification of compounds identified in PGE.

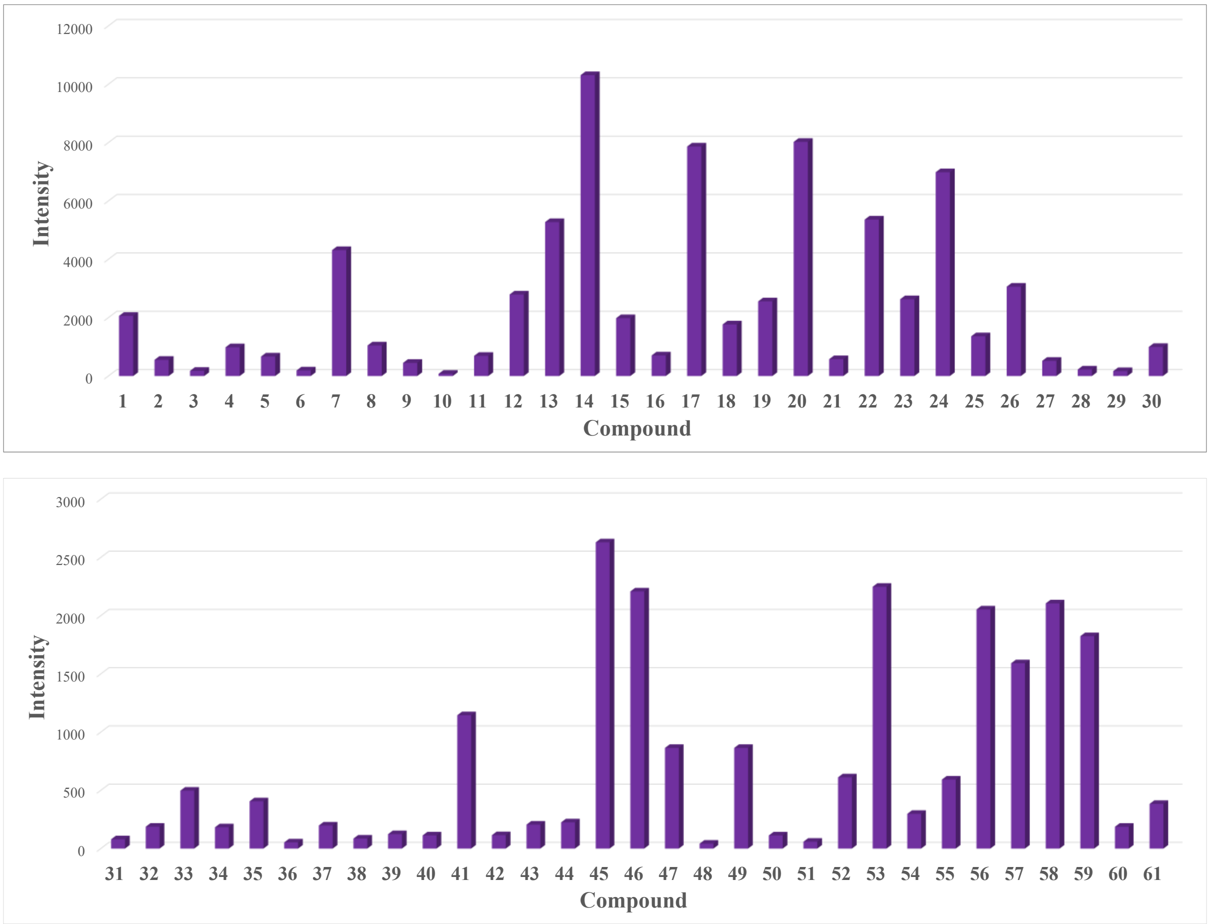

Figure S7. Composition of compound 1−61 identified from the PGE extract.

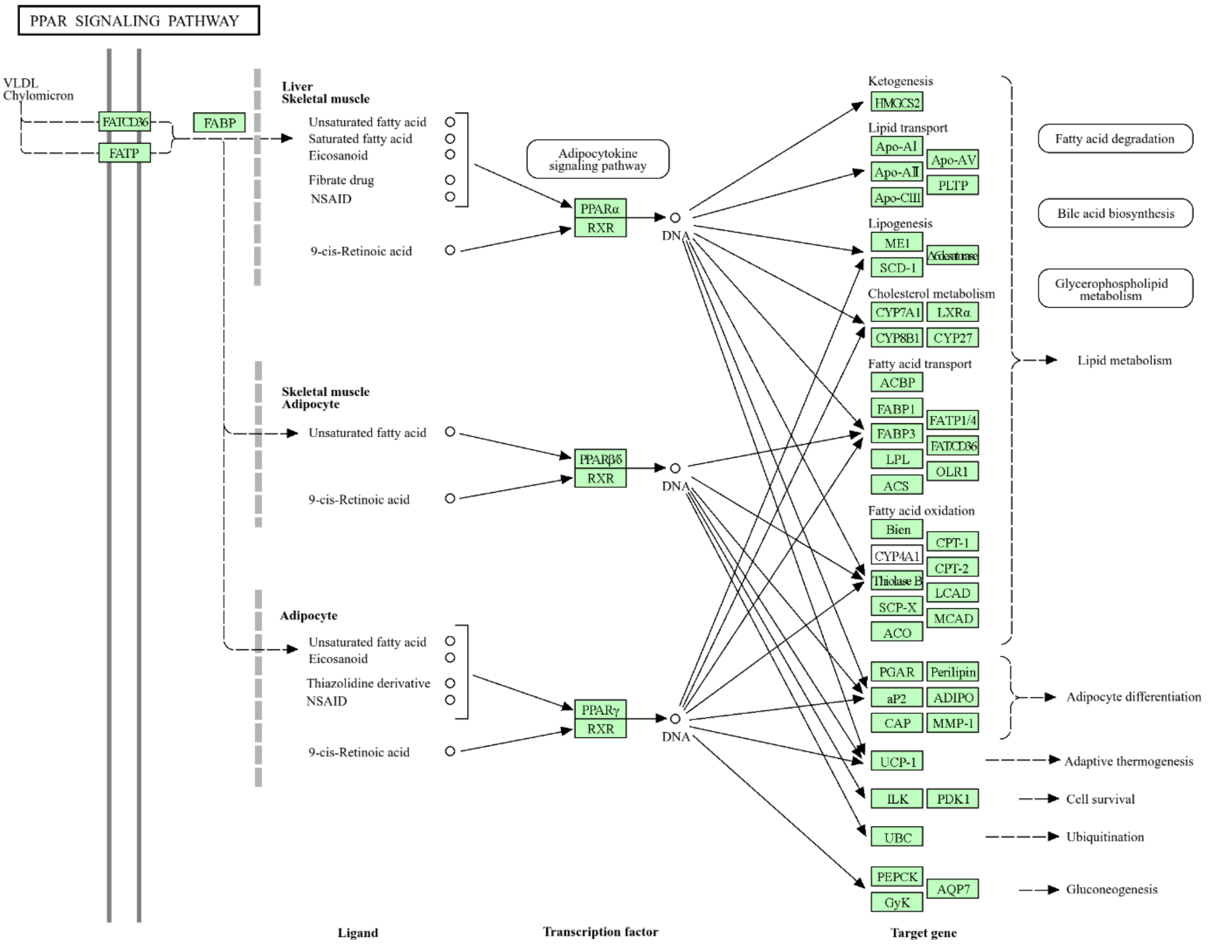

Figure S8. Enrichment of obesity-related targets in the PPAR signaling pathway. This pathway involves various ligands, transcription factors, and target genes that play key roles in obesity and related metabolic disorders.

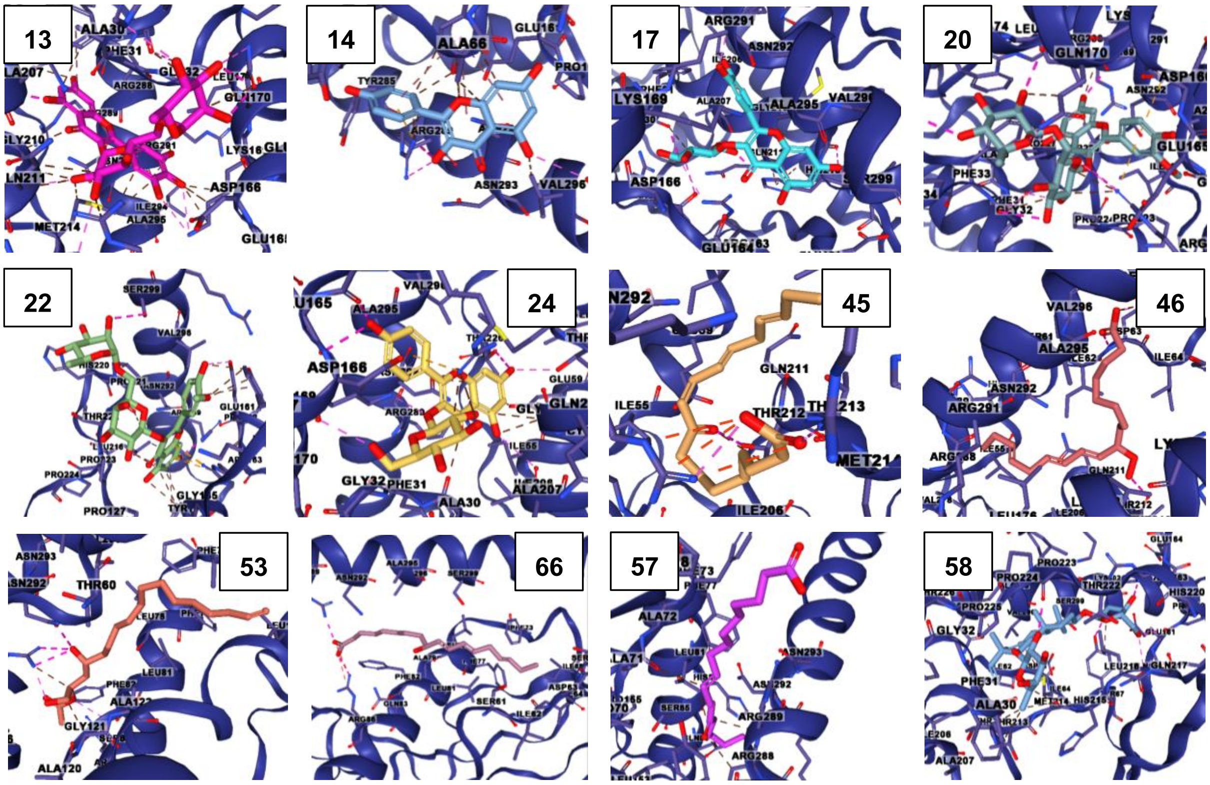

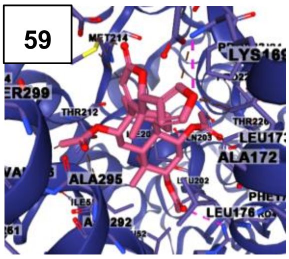

Ligand	Score (kcal/mol)	Ligand	Scores (kcal/mol)	Ligand	Score (kcal/mol)	Ligand	Score (kcal/mol)
13	−3.6	22	−5.0	53	−4.8	59	−5.6
14	−2.4	24	−3.5	56	−4.9		
17	−4.1	45	−4.9	57	−4.4	Lico A (Native ligand)	−6.3
20	−5.2	46	−5.4	58	−7.4	Protein (PDB ID: 1NWQ)	

Figure S9. 3D Interactions of compounds 13, 14, 17, 20, 22, 24, 45, 46, 53, 56, 57, 58, and 59 from PGE with amino acid when they were docked into C/EPBα protein (**PDB ID: 1NWQ**).

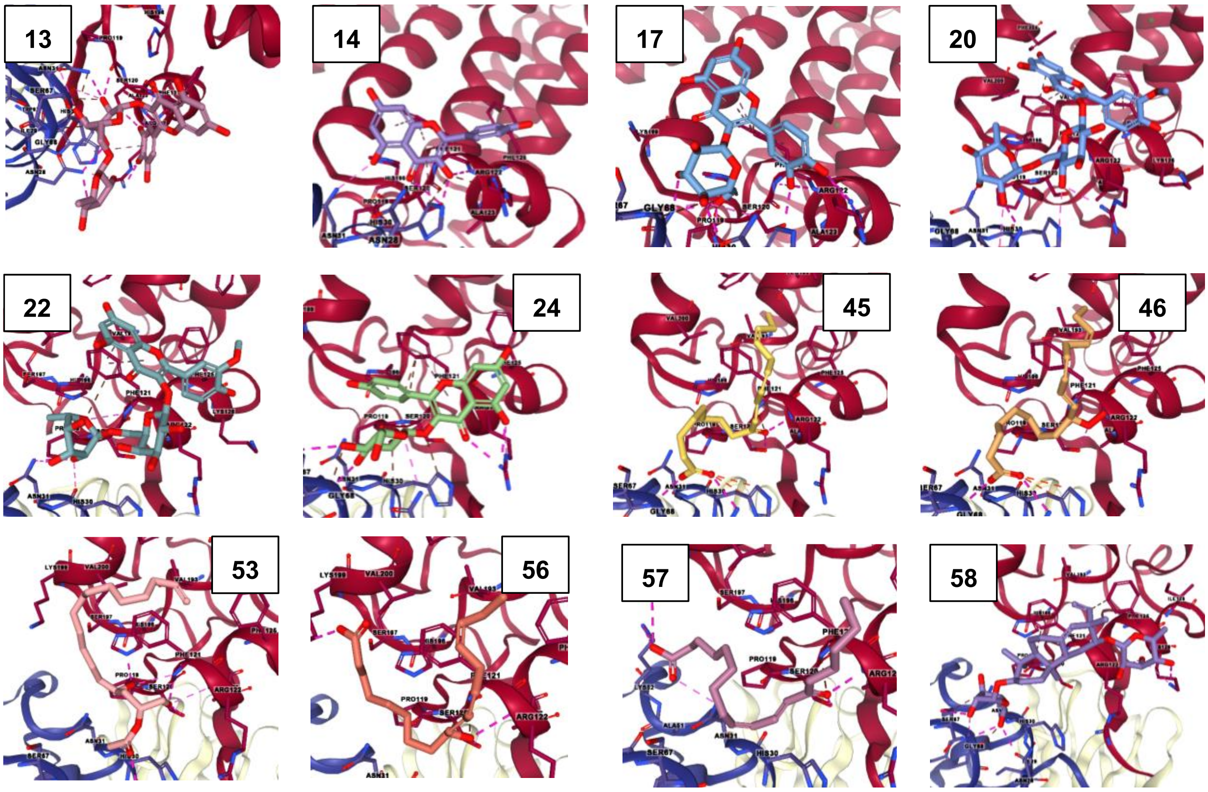

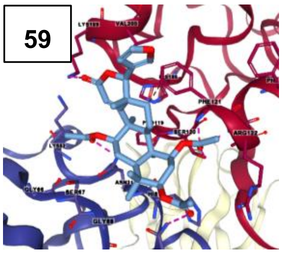

Ligand	Score (kcal/mol)	Ligand	Score (kcal/mol)	Ligand	Score (kcal/mol)	Ligand	Score (kcal/mol)
13	–5.1	22	–5.0	53	–4.4	59	–4.1
14	–3.4	24	–3.7	56	–3.6		
17	–4.2	45	–4.2	57	–3.4	Native ligand	–1.5
20	–4.1	46	–3.9	58	–4.1	Protein (PDB ID: 6KS0)	

Figure S10. 3D Interactions of compounds 13, 14, 17, 20, 22, 24, 45, 46, 53, 56, 57, 58, and 59 from PGE with amino acid when they were docked into adiponectin protein (**PDB ID: 6KS0**).
